# HOSVD-Based Algorithm for Weighted Tensor Completion

**DOI:** 10.3390/jimaging7070110

**Published:** 2021-07-07

**Authors:** Zehan Chao, Longxiu Huang, Deanna Needell

**Affiliations:** Department of Mathematics, University of California, Los Angeles, CA 90095, USA; zchao3@math.ucla.edu (Z.C.); deanna@math.ucla.edu (D.N.)

**Keywords:** HOSVD decomposition, tensor completion, weighted tensor

## Abstract

Matrix completion, the problem of completing missing entries in a data matrix with low-dimensional structure (such as rank), has seen many fruitful approaches and analyses. Tensor completion is the tensor analog that attempts to impute missing tensor entries from similar low-rank type assumptions. In this paper, we study the tensor completion problem when the sampling pattern is deterministic and possibly non-uniform. We first propose an efficient weighted Higher Order Singular Value Decomposition (HOSVD) algorithm for the recovery of the underlying low-rank tensor from noisy observations and then derive the error bounds under a properly weighted metric. Additionally, the efficiency and accuracy of our algorithm are both tested using synthetic and real datasets in numerical simulations.

## 1. Introduction

In many data-rich domains such as computer vision, neuroscience, and social networks, tensors have emerged as a powerful paradigm for handling the data deluge. In recent years, tensor analysis has gained more and more attention. To a certain degree, tensors can be viewed as the generalization of matrices to higher dimensions, and thus multiple questions from matrix analysis extend naturally to tensors. Similar to matrix decomposition, the problem of tensor decomposition (decomposing an input tensor into several less complex components) has been widely studied both in theory and application (see e.g., [1,2,3]). Thus far, the problem of low-rank tensor completion, which aims to complete missing or unobserved entries of a low-rank tensor, is one of the most actively studied problems (see e.g., [4,5,6,7]). It is noteworthy that, as caused by various unpredictable or unavoidable reasons, multidimensional datasets are commonly raw and incomplete, and thus often only a small subset of entries of tensors are available. It is, therefore, natural to address the above issue using tensor completion in modern data-driven applications, in which data are naturally represented as a tensor, such as image/video inpainting [5,8], link-prediction [9], and recommendation systems [10], to name a few.

In the past few decades, the matrix completion problem, which is a special case of tensor completion, has been extensively studied. In matrix completion, there are mature algorithms [11], theoretical foundations [12,13,14] and various applications [15,16,17,18] that pave the way for solving the tensor completion problem in high-order tensors. Recently, Foucart et al. [19] proposed a simple algorithm for matrix completion for general deterministic sampling patterns, and raised the following questions: given a deterministic sampling pattern Ω and corresponding (possibly noisy) observations of the matrix entries, what type of recovery error can we expect? In what metric? How can we efficiently implement recovery? These were investigated in [19] by introducing an appropriate *weighted* error metric for matrix recovery of the form $∥H⊡\left(\hat{M}-M\right){∥}_{F}$, where *M* is the true underlying low-rank matrix, $\hat{M}$ refers to the recovered matrix, and *H* is a best rank-1 matrix approximation for the sampling pattern Ω. In this regard, similar questions arise for the problem of tensor completion with deterministic sampling patterns. Unfortunately, as is often the case, moving from the matrix setting to the tensor setting presents non-trivial challenges, and notions such as *rank* and SVD need to be re-defined and re-evaluated. We address these extensions for the completion problem here.

Motivated by the matrix case, we propose an appropriate *weighted* error metric for tensor recovery of the form $∥H⊡\left(\hat{T}-T\right){∥}_{F}$, where T is the true underlying low-rank tensor, $\hat{T}$ is the recovered tensor, and H is an appropriate weight tensor. For the existing work, the error is only limited to the form $∥\hat{T}{-T∥}_{F}$, which corresponds to the case that all the entries of H are 1, where H can be considered to be a CP rank-1 tensor. It motivates us to rephrase the questions mentioned above as follows.

**Main questions.** Given a sampling pattern Ω, and noisy observations T+Z on Ω, for what rank-one weight tensor H can we efficiently find a tensor $\hat{T}$ so that $∥H⊡\left(\hat{T}-T\right){∥}_{F}$ is small compared to ${∥H∥}_{F}$? And how can we efficiently find such weight tensor H, or determine that a fixed H has this property?

### 1.1. Contributions

Our main goal is to provide an algorithmic tool, theoretical analysis, and numerical results that address the above questions. In this paper, we propose a simple weighted Higher Order Singular Value Decomposition (HOSVD) method. Before we implement the weighted HOSVD algorithm, we first appropriately approximate the sampling pattern Ω with a rank one tensor H. We can achieve high accuracy if $∥H-H^{\left(-1\right)}⊡1_{\Omega }{∥}_{F}$ is small, where $H^{\left(-1\right)}$ denotes the element-wise inverse. Finally, we present empirical results on synthetic and real datasets. The simulation results show that when the sampling pattern is non-uniform, the use of weights in the weighted HOSVD algorithm is essential, and the results of the weighted HOSVD algorithm can provide a very good initialization for the total variation minimization algorithm which can dramatically reduce the iterative steps without lose the accuracy. In doing so, we extend the weighted matrix completion results of [19] to the tensor setting.

### 1.2. Organization

The paper is organized as follows. In Section 2, we give a brief review of related work and concepts for tensor analysis, instantiate notations, and state the tensor completion problem under study. Our main results are stated in Section 3 and the proofs are provided in Appendix A and Appendix B. The numerical results are provided and discussed in Section 4.

## 2. Related Work, Background, and Problem Statement

In this section, we give a brief overview of the works that are related to ours, introduce some necessary background information about tensors, and finally give a formal statement of tensor completion problem under study. The related work can be divided into two lines: that based on matrix completion problems, which leads to a discussion of weighted matrix completion and related work, and that based on tensor analysis, in which we focus on CP and Tucker decompositions.

### 2.1. Matrix Completion

The matrix completion problem is to determine a complete $d_{1}\times d_{2}$ matrix *M* from its partial entries on a subset $\Omega \subseteq \left[d_{1}\right]\times \left[d_{2}\right]$. We use 1${}_{\Omega }$ to denote the matrix whose entries are 1 on Ω and 0 elsewhere so that the entries of $M_{\Omega }=1_{\Omega }⊡M$ are equal to those of the matrix *M* on Ω, and are equal to 0 elsewhere, where ⊡ denotes the Hadamard product. There are various works that aim to understand matrix completion with respect to the sampling pattern Ω. For example, the works in [20,21,22] relate the sampling pattern Ω to a graph whose adjacency matrix is given by $1_{\Omega }$ and show that as long as the sampling pattern Ω is suitably close to an expander, efficient recovery is possible when the given matrix *M* is sufficiently incoherent. Mathematically, the task of understanding when there exists a unique low-rank matrix *M* that can complete $M_{\Omega }$ as a function of the sampling pattern Ω is very important. In [23], the authors give conditions on Ω under which there are only finitely many low-rank matrices that agree with $M_{\Omega }$, and the work of [24] gives a condition under which the matrix can be locally uniquely completed. The work in [25] generalized the results of [23,24] to the setting where there is sparse noise added to the matrix. The works [26,27] study when rank estimation is possible as a function of a deterministic pattern Ω. Recently, [28] gave a combinatorial condition on Ω that characterizes when a low-rank matrix can be recovered up to a small error in the Frobenius norm from observations in Ω and showed that nuclear minimization will approximately recover *M* whenever it is possible, where the *nuclear norm* of *M* is defined as ${∥M∥}_{*}:=\sum_{i=1}^{r}\sigma_{i}$ with $\sigma_{1},\cdots ,\sigma_{r}$ the non-zero singular values of *M*.

All the works mentioned above are in the setting where recovery of the entire matrix is possible, but in many cases full recovery is impossible. Ref. [29] uses an algebraic approach to answer the question of when an individual entry can be completed. There are many works (see e.g., [30,31]) that introduce a weight matrix for capturing the recovery results of the desired entries. The work [21] shows that, for any weight matrix, *H*, there is a deterministic sampling pattern Ω and an algorithm that returns $\hat{M}$ using the observation $M_{\Omega }$ such that $∥H⊡\left(\hat{M}-M\right){∥}_{F}$ is small. The work [32] generalizes the algorithm in [21] to find the “simplest” matrix that is correct on the observed entries. Succinctly, their works give a way of measuring which deterministic sampling patterns, Ω, are “good” with respect to a weight matrix *H*. In contrast to these two works, [19] is interested in the problem of whether one can find a weight matrix *H* and create an efficient algorithm to find an estimate $\hat{M}$ for an underlying low-rank matrix *M* from a sampling pattern Ω and noisy samples $M_{\Omega }+Z_{\Omega }$ such that $∥H⊡\left(\hat{M}-M\right){∥}_{F}$ is small.

In particular, one of our theoretical results is that we generalize the upper bounds for weighted recovery of low-rank matrices from deterministic sampling patterns in [19] to the upper bound of tensor weighted recovery. The details of the connection between our result and the matrix setting result in [19] is discussed in Section 3.

### 2.2. Tensor Completion Problem

Tensor completion is the problem of filling in the missing elements of partially observed tensors. Similar to the matrix completion problem, *low rankness* is often a necessary hypothesis to restrict the degrees of freedom of the missing entries for the tensor completion problem. Since there are multiple definitions of the rank of a tensor, this completion problem has several variations.

The most common tensor completion problems [5,33] may be summarized as follows (we will define the different ranks subsequently, see further on in this section).

**Definition** **1** (Low-rank tensor completion (LRTC) [7]). *Given a low-rank (CP rank, Tucker rank, or other ranks) tensor T and sampling pattern Ω, the low-rank completion of T is given by the solution of the following optimization problem:*

(1) $$\begin{array}{ccc} & & \min_{X}\;{\mathrm{rank}}_{*}\left(X\right)\\ & & \mathrm{subject}\;\mathrm{to}\;X_{\Omega }=T_{\Omega }, \end{array}$$

where ${\mathrm{rank}}_{*}$ denotes the specific tensor rank assumed at the beginning.

In the literature, there are many variants of LRTC but most of them are based on the following questions:

(1) What type of the rank should one use (see e.g., [34,35,36])?
(2) Are there any other restrictions based on the observations that one can assume (see e.g., [5,37,38])?
(3) Under what conditions can one expect to achieve a unique and exact completion (see e.g., [34])?

In the rest of this section, we instantiate some notations and review basic operations and definitions related to tensors. Then some tensor decomposition-based algorithms for tensor completion are stated. Finally, a formal problem statement under study will be presented.

#### 2.2.1. Preliminaries and Notations

Tensors, matrices, vectors, and scalars are denoted in different typeface for clarity below. In the sequel, calligraphic boldface capital letters are used for tensors, capital letters are used for matrices, lower boldface letters for vectors, and regular letters for scalars. The set of the first *d* natural numbers is denoted by [d]:={1,⋯,d}. Let $X\in R^{d_{1}\times \cdots \times d_{n}}$ and α∈R, $X^{\left(\alpha \right)}$ represents the element-wise power operator, i.e., ${\left(X^{\left(\alpha \right)}\right)}_{i_{1}\cdots i_{n}}=X_{i_{1}\cdots i_{n}}^{\alpha }$. $1_{\Omega }\in R^{d_{1}\times \cdots \times d_{n}}$ denotes the tensor with 1 on Ω and 0 otherwise. We use X≻0 to denote the tensor with $X_{i_{1}\cdots i_{n}}>0$ for all $i_{1},\cdots ,i_{n}$. Moreover, we say that Ω∼W if the entries of X are sampled randomly with the sampling set Ω such that $(i_{1},\cdots ,i_{n})\in \Omega$ with probability $W_{i_{1}\cdots i_{n}}$. We include here some basic notions relating to tensors, and refer the reader to e.g., [2] for a more thorough survey.

**Definition** **2** (Tensor). *A tensor is a multidimensional array. The dimension of a tensor is called the order (also called the mode). The space of real tensors of order n and size $d_{1}\times \cdots \times d_{n}$ is denoted as $R^{d_{1}\times \cdots \times d_{n}}$. The elements of a tensor $X\in R^{d_{1}\times \cdots \times d_{n}}$ are denoted by $X_{i_{1}\cdots i_{n}}$.*

An *n*-order tensor X can be matricized in *n* ways by unfolding it along each of the *n* modes. The definition for the matricization of a given tensor is stated below.

**Definition** **3** (Matricization/unfolding of a tensor). *The mode-k matricization/unfolding of tensor $X\in R^{d_{1}\times \cdots \times d_{n}}$ is the matrix, which is denoted as $X_{\left(k\right)}\in R^{d_{k}\times \prod_{j\neq k}d_{j}}$, whose columns are composed of all the vectors obtained from X by fixing all indices except for the k-th dimension. The mapping $X\mapsto X_{\left(k\right)}$ is called the mode-k unfolding operator.*

**Example** **1.** *Let $X\in R^{3\times 4\times 2}$ with the following frontal slices:*

$$X_{1}=\left[\begin{array}{cccc} 1 & 4 & 7 & 10\\ 2 & 5 & 8 & 11\\ 3 & 6 & 9 & 12 \end{array}\right]\;\;\;\;X_{2}=\left[\begin{array}{cccc} 13 & 16 & 19 & 22\\ 14 & 17 & 20 & 23\\ 15 & 18 & 21 & 24 \end{array}\right],$$

*then the three mode-n matricizations are*

$$\begin{array}{ccc} X_{\left(1\right)} & = & \left[\begin{array}{cccccccc} 1 & 4 & 7 & 10 & 13 & 16 & 19 & 22\\ 2 & 5 & 8 & 11 & 14 & 17 & 20 & 23\\ 3 & 6 & 9 & 12 & 15 & 18 & 21 & 24 \end{array}\right],\\ X_{\left(2\right)} & = & \left[\begin{array}{cccccc} 1 & 2 & 3 & 13 & 14 & 15\\ 4 & 5 & 6 & 16 & 17 & 18\\ 7 & 8 & 9 & 19 & 20 & 21\\ 10 & 11 & 12 & 22 & 23 & 24 \end{array}\right],\\ X_{\left(3\right)} & = & \left[\begin{array}{ccccccc} 1 & 2 & 3 & \cdots & 10 & 11 & 12\\ 13 & 14 & 15 & \cdots & 22 & 23 & 24 \end{array}\right]. \end{array}$$

**Definition** **4** (Folding operator). *Suppose that X is a tensor. The mode-k folding operator of a matrix $M=X_{\left(k\right)}$, denoted as ${\mathrm{fold}}_{k}\left(M\right)$, is the inverse operator of the unfolding operator.*

**Definition** **5** (∞-norm). *Given $X\in R^{d_{1}\times \cdots \times d_{n}}$, the norm ${∥X∥}_{\infty }$ is defined as*

$${∥X∥}_{\infty }=\max_{i_{1},\cdots ,i_{n}}\left|X_{i_{1}\cdots i_{n}}\right|.$$

*The unit ball under the ∞-norm is denoted by B${}_{\infty }$.*

**Definition** **6** (Frobenius norm). *The Frobenius norm for a tensor $X\in R^{d_{1}\times \cdots \times d_{n}}$ is defined as*

$${∥X∥}_{F}=\sqrt{\sum_{i_{1},\cdots ,i_{n}}X_{i_{1}\cdots i_{n}}^{2}}.$$

**Definition** **7** (Max-norm for matrix). *Given $X\in R^{d_{1}\times d_{2}}$, the max-norm for X is defined as*

$${∥X∥}_{max}=\min_{X=UV^{T}}{∥U∥}_{2,\infty }{∥V∥}_{2,\infty }.$$

**Definition** **8** (Product operations). * *

- *Outer product: Let $a_{1}\in R^{d_{1}},\cdots ,a_{n}\in R^{d_{n}}$. The outer product among these n vectors is a tensor $X\in R^{d_{1}\times \cdots \times d_{n}}$ defined as:*
  $$X=a_{1}⊗\cdots ⊗a_{n},\;\;X_{i_{1},\cdots ,i_{n}}=\prod_{k=1}^{n}a_{k}\left(i_{k}\right).$$
  *The tensor $X\in R^{d_{1}\times \cdots \times d_{n}}$ is of rank one if it can be written as the outer product of n vectors.*
- *Kronecker product of matrices: The Kronecker product of $A\in R^{I\times J}$ and $B\in R^{K\times L}$ is denoted by A⊗B. The result is a matrix of size (KI)×(JL) defined by*
  $$\begin{array}{ccc} A⊗B & = & \left[\begin{array}{cccc} A_{11}B & A_{12}B & \cdots & A_{1J}B\\ A_{21}B & A_{22}B & \cdots & A_{2J}B\\ \vdots & \vdots & \ddots & \vdots \\ A_{I1}B & A_{I2}B & \cdots & A_{IJ}B \end{array}\right]. \end{array}$$
- *Khatri-Rao product: Given matrices $A\in R^{d_{1}\times r}$ and $B\in R^{d_{2}\times r}$, their Khatri-Rao product is denoted by A⊙B. The result is a matrix of size $(d_{1}d_{2})\times r$ defined by*
  $$A⊙B=\left[\begin{array}{ccc} a_{1}⊗b_{1} & \cdots & a_{r}⊗b_{r} \end{array}\right],$$
  *where $a_{i}$ and $b_{i}$ stand for the i-th column of A and B respectively.*
- *Hadamard product: Given $X,Y\in R^{d_{1}\times \cdots \times d_{n}}$, their Hadamard product $X⊡Y\in R^{d_{1}\times \cdots \times d_{n}}$ is defined by element-wise multiplication, i.e.,*
  $${\left(X⊡Y\right)}_{i_{1}\cdots i_{n}}=X_{i_{1}\cdots i_{n}}Y_{i_{1}\cdots i_{n}}.$$
- *Mode-k product: Let $X\in R^{d_{1}\times \cdots \times d_{n}}$ and $U\in R^{d_{k}\times J}$, the multiplication between X on its mode-k with U is denoted as $Y=X\times_{k}U$ with*
  $$Y_{i_{1},\cdots ,i_{k-1},j,i_{k+1},\cdots ,i_{n}}=\sum_{s=1}^{d_{k}}X_{i_{1},\cdots ,i_{k-1},s,i_{k+1},\cdots ,i_{n}}U_{s,j}.$$

**Definition** **9** (Tensor (CP) rank [1,39]). *The (CP) rank of a tensor X, denoted rank(X), is defined as the smallest number of rank-1 tensors that generate X as their sum. We use $K_{r}$ to denote the cone of rank-r tensors.*

Given ${}^{k}M\in R^{d_{k}\times r}$, we use $〚{}^{1}M,\cdots ,{}^{n}M〛$ to denote the CP representation of tensor X, i.e.,

$$X=\sum_{j=1}^{r}\left({}^{1}M\left(:,j\right)⊗\cdots ⊗{}^{n}M\left(:,j\right)\right),$$

where M(:,j) means the *j*-th column of the matrix *M*.

Different from the case of matrices, the rank of a tensor is not presently well understood. Additionally, the task of computing the rank of a tensor is an NP-hard problem [40]. Next we introduce an alternative definition of the rank of a tensor, which is easy to compute.

**Definition** **10** (Tensor Tucker rank [39]). *Let $X\in R^{d_{1}\times \cdots \times d_{n}}$. The tuple $\left(r_{1},\cdots ,r_{n}\right)\in N^{n}$ is called the Tucker rank of the tensor X, where $r_{k}=\mathrm{rank}\left(X_{\left(k\right)}\right)$. We use $K_{r}$ to denote the cone of tensors with Tucker rank r.*

Tensor decompositions are powerful tools for extracting meaningful, latent structures in heterogeneous, multidimensional data (see e.g., [2]). In this paper, we focus on two most widely used decomposition methods: CP and HOSVD. For more comprehensive introduction, readers are referred to [2,41,42].

#### 2.2.2. CP-Based Method for Tensor Completion

The CP decomposition was first proposed by Hitchcock [1] and further discussed in [43]. The formal definition of the CP decomposition is the following.

**Definition** **11** (CP decomposition). *Given a tensor $X\in R^{d_{1}\times \cdots \times d_{n}}$, its CP decomposition is an approximation of n loading matrices $A_{k}\in R^{d_{k}\times r}$, k=1,⋯,n, such that*

$$X\approx 〚A_{1},\cdots ,A_{n}〛=\sum_{i=1}^{r}A_{1}\left(:,i\right)⊗\cdots ⊗A_{n}\left(:,i\right),$$

*where r is a positive integer denoting an upper bound of the rank of X and $A_{k}\left(:,i\right)$ is the i-th column of matrix $A_{k}$. If we unfold X along its k-th mode, we have*

$$X_{\left(k\right)}\approx A_{k}{\left(A_{1}⊙\ldots ⊙A_{k-1}⊙A_{k+1}⊙\cdots ⊙A_{n}\right)}^{T}.$$

Here the ≈ sign means that the algorithm should find an optimal $\hat{X}$ with the given rank such that the distance between the low-rank approximation and the original tensor, $∥X-\hat{X}{∥}_{F}$, is minimized.

Given an observation set Ω, the main idea to implement tensor completion for a low-rank tensor T is to conduct imputation based on the equation

$$X=T_{\Omega }+{\hat{X}}_{\Omega^{c}},$$

where $\hat{X}=〚A_{1},\cdots ,A_{n}〛$ is the interim low-rank approximation based on the CP decomposition, X is the recovered tensor used in next iteration for decomposition, and $\Omega^{c}=\left\{\left(i_{1},\cdots ,i_{n}\right):1\leq i_{k}\leq d_{k}\right\}\setminus \Omega$. For each iteration, we usually estimate the matrices $A_{k}$ using the alternating least squares optimization method (see e.g., [44,45,46]).

#### 2.2.3. HOSVD-Based Method for Tensor Completion

The Tucker decomposition was proposed by Tucker [47] and further developed in [48,49].

**Definition** **12** (Tucker decomposition). *Given an n-order tensor X, its Tucker decomposition is defined as an approximation of a core tensor $C\in R^{r_{1}\times \cdots \times r_{n}}$ multiplied by n factor matrices $A_{k}\in R^{d_{k}\times r_{k}}$, k=1,⋯,n along each mode, such that*

$$X\approx C\times_{1}A_{1}\times_{2}\cdots \times_{n}A_{n}=〚C;A_{1},\cdots ,A_{n}〛,$$

*where $r_{k}$ is a positive integer denoting an upper bound of the rank of the matrix $X_{\left(k\right)}$.*

*If we unfold X along its k-th mode, we have*

$$X_{\left(k\right)}\approx A_{k}C_{\left(k\right)}{\left(A_{1}⊗\cdots ⊗A_{k-1}⊗A_{k+1}⊗\cdots ⊗A_{n}\right)}^{T}$$

Tucker decomposition is a widely used tool for tensor completion. To implement Tucker decomposition, one popular method is called the higher-order SVD (HOSVD) [47]. The main idea of HOSVD is:

1. Unfold X along mode *k* to obtain matrix $X_{\left(k\right)}$;
2. Find the economic SVD decomposition of $X_{\left(k\right)}={}^{k}U{}^{k}\Sigma {}^{k}V^{T}$;
3. Set $A_{k}$ to be the first $r_{k}$ columns of ${}^{k}U$;
4. $C=X\times_{1}A_{1}^{T}\times_{2}\cdots \times_{n}A_{n}^{T}$.

If we want to find a Tucker rank $r=[r_{1},\cdots ,r_{n}]$ approximation for the tensor X via HOSVD process, we just replace $A_{k}$ by the first $r_{k}$ columns of $U_{k}$.

#### 2.2.4. Tensor Completion Problem under Study

In our setting, it is supposed that T is an unknown tensor in $K_{r}\cap \beta B_{\infty }$ or $K_{r}\cap \beta B_{\infty }$. Fix a sampling pattern $\Omega \subseteq \left[d_{1}\right]\times \cdots \times \left[d_{n}\right]$ and the weight tensor W. Our goal is to design an algorithm that gives provable guarantees for a worst-case T, even if it is adapted to Ω.

In our algorithm, the observed data are $T_{\Omega }+Z_{\Omega }=1_{\Omega }⊡\left(T+Z\right)$, where $Z_{i_{1}\cdots i_{n}}∼N\left(0,\sigma^{2}\right)$ are i.i.d. Gaussian random variables. From the observations, the goal is to learn something about T. In this paper, instead of measuring our recovered results with the underlying true tensor in a standard Frobenius norm $∥T-\hat{T}{∥}_{F}$, we are interested in learning T using a *weighted* Frobenius norm, i.e., to develop an efficient algorithm to find $\hat{T}$ so that

$${∥W^{\left(1/2\right)}⊡\left(T-\hat{T}\right)∥}_{F}$$

is as small as possible for some weight tensor W. When measuring the weighted error, it is important to normalize appropriately to understand the meaning of the error bounds. In our results, we always normalize the error bounds by ${∥W^{\left(1/2\right)}∥}_{F}$. It is noteworthy that

$$\begin{array}{ccc} \frac{{∥W^{\left(1/2\right)}⊡\left(T-\hat{T}\right)∥}_{F}}{{∥W^{\left(1/2\right)}∥}_{F}} & = & {\left(\sum_{i_{1},\cdots ,i_{n}}\frac{W_{i_{1}\cdots i_{n}}}{\sum_{i_{1},\cdots ,i_{n}}W_{i_{1},\cdots ,i_{n}}}{\left(T_{i_{1}\cdots i_{n}}-{\hat{T}}_{i_{1}\cdots i_{n}}\right)}^{2}\right)}^{1/2}, \end{array}$$

which gives a weighted average of the per entry squared error. Generally, our problem can be formally stated below.
**Problem**: Weighted Universal Tensor Completion **Parameters:** Dimensions $d_{1},\cdots ,d_{n}$;A sampling pattern $\Omega \subseteq \left[d_{1}\right]\times \cdots \times \left[d_{n}\right]$;Parameters σ,β>0, *r* or $r=[r_{1}\;\cdots \;r_{n}]$;A rank-1 weight tensor $W\in R^{d_{1}\times \cdots \times d_{n}}$ so that $W_{i_{1}\cdots i_{n}}>0$ for all $i_{1},\cdots ,i_{n}$;A set *K* (e.g., $K_{r}\cap \beta B_{\infty }$ or $K_{r}\cap \beta B_{\infty }$). **Goal:** Design an efficient algorithm A with the following guarantees:
A takes as input entries $T_{\Omega }+Z_{\Omega }$ so that $Z_{i_{1}\cdots i_{n}}∼N\left(0,\sigma^{2}\right)$ are i.i.d.;A runs in polynomial time;With high probability over the choice of Z, A returns an estimate $\hat{T}$ of T so that
$$\frac{{∥W^{\left(1/2\right)}⊡\left(T-\hat{T}\right)∥}_{F}}{{∥W^{\left(1/2\right)}∥}_{F}}\leq \delta$$
for all T∈K, where δ depends on the problem parameters.

**Remark** **1** (Strictly positive W). *The requirement that $W_{i_{1}\cdots i_{n}}$ is strictly greater than zero is a generic condition. In fact, if $W_{i_{1}\cdots i_{n}}=0$ for some $\left(i_{1},\cdots ,i_{n}\right)$, some mode k with index $i_{k}$ of W is zero, then we can reduce the problem to a smaller one by ignoring that mode k with index $i_{k}$.*

## 3. Main Results

In this section, we state informal versions of our main results. With fixed sampling pattern Ω and weight tensor W, we can find $\hat{T}$ by solving the following optimization problem:

(2) $$\hat{T}=W^{\left(-1/2\right)}⊡\underset{\mathrm{rank}\;(X)=r}{\mathrm{argmin}}{∥X-W^{\left(-1/2\right)}⊡Y_{\Omega }∥}_{F},$$

or

(3) $$\hat{T}=W^{\left(-1/2\right)}⊡\underset{\mathrm{Tucker}-\mathrm{rank}\;(X)=r}{\mathrm{argmin}}{∥X-W^{\left(-1/2\right)}⊡Y_{\Omega }∥}_{F},$$

where $Y_{\Omega }\in R^{d_{1}\times \cdots \times d_{n}}$ with

$$Y_{\Omega }\left(i_{1},\cdots ,i_{n}\right)=\left\{\begin{array}{cc} T_{i_{1}\cdots i_{n}}+Z_{i_{1}\cdots i_{n}} & \;\mathrm{if}\;(i_{1},\cdots ,i_{n})\in \Omega \\ 0 & \;\mathrm{if}\;(i_{1},\cdots ,i_{n})\notin \Omega \end{array}\right..$$

It is known that solving (2) is NP-hard [40]. However, there are some polynomial time algorithms to find approximate solutions for (2) such that the approximation is (empirically) close to the actual solution of (2) in terms of the Frobenius norm. In our numerical experiments, we solve (2) via the CP-ALS algorithm [43]. To solve (3), we use the HOSVD process [48]. Assume that T has Tucker rank $r=[r_{1},\cdots ,r_{n}]$. Let

$${\hat{A}}_{i}=\underset{\mathrm{rank}\;\left(A\right)=r_{i}}{\mathrm{argmin}}{∥A-{\left(W^{\left(-1/2\right)}⊡Y_{\Omega }\right)}_{\left(i\right)}∥}_{2}$$

and set ${\hat{U}}_{i}$ to be the left singular vector matrix of ${\hat{A}}_{i}$. Then the estimated tensor is of the form

$$\hat{T}=W^{\left(-1/2\right)}⊡(\left(W^{\left(-1/2\right)}⊡Y_{\Omega }\right)\times_{1}{\hat{U}}_{1}{\hat{U}}_{1}^{T}\times_{2}\cdots \times_{n}{\hat{U}}_{n}{\hat{U}}_{n}^{T}.$$

In the following, we call this the weighted HOSVD algorithm.

### 3.1. General Upper Bound

Suppose that the optimal solution $\hat{T}$ for (2) or (3) $\hat{T}$ can be found, we would like to give an upper bound estimations for $∥W^{\left(1/2\right)}⊡\left(T-\hat{T}\right){∥}_{F}$ with some proper weight tensor W.

**Theorem** **1.** *Let $W=w_{1}⊗\cdots ⊗w_{n}\in R^{d_{1}\times \cdots \times d_{n}}$ have strictly positive entries, and fix $\Omega \subseteq \left[d_{1}\right]\times \cdots \times \left[d_{n}\right]$. Suppose that $T\in R^{d_{1}\times \cdots \times d_{n}}$ has rank r for problem (2) or Tucker rank $r=[r_{1},\cdots ,r_{n}]$ for problem (3), and let $\hat{T}$ be the optimal solutions for (2) or (3). Suppose that $Z_{i_{1}\cdots i_{n}}∼N\left(0,\sigma^{2}\right)$. Then with probability at least $1-2^{-|\Omega |/2}$ over the choice of Z,*

$${∥W^{\left(1/2\right)}⊡\left(T-\hat{T}\right)∥}_{F}\leq 2{∥T∥}_{\infty }{∥W^{\left(1/2\right)}-W^{\left(-1/2\right)}⊡1_{\Omega }∥}_{F}+4\sigma \mu \sqrt{\left|\Omega |\log(2\right)},$$

*Recall here, ${\left(W^{\left(1/2\right)}\right)}_{i_{1}\cdots i_{n}}=W_{i_{1}\cdots i_{n}}^{\left(1/2\right)}$ and ${\left(W^{\left(-1/2\right)}\right)}_{i_{1}\cdots i_{n}}=W_{i_{1}\cdots i_{n}}^{\left(-1/2\right)}$ as defined in Section 2.2.1 and $\mu^{2}=\max_{(i_{1},\cdots ,i_{n})\in \Omega }\frac{1}{W_{i_{1}\cdots i_{n}}}$.*

Notice that the upper bound in Theorem 1 is for the optimal output $\hat{T}$ for problems (2) and (3), which is general. However, the upper bound in Theorem 1 contains no rank information of the underlying tensor T. To introduce the rank information of the underlying tensor T, we restrict our analysis for Problem (3) by considering the HOSVD process in the sequel.

### 3.2. Results for Weighted HOSVD Algorithm

In this section, we begin by giving a general upper bound for the weighted HOSVD algorithm.

#### 3.2.1. General Upper Bound for Weighted HOSVD

**Theorem** **2** (Informal, see Theorem A1). *Let $W=w_{1}⊗\cdots ⊗w_{n}\in R^{d_{1}\times \cdots \times d_{n}}$ have strictly positive entries, and fix $\Omega \subseteq \left[d_{1}\right]\times \cdots \times \left[d_{n}\right]$. Suppose that $T\in R^{d_{1}\times \cdots \times d_{n}}$ has Tucker rank $r=[r_{1},\cdots ,r_{n}]$. Suppose that $Z_{i_{1}\cdots i_{n}}∼N\left(0,\sigma^{2}\right)$ and let $\hat{T}$ be the estimate of the solution of (3) via the HOSVD process. Then*

$$\begin{array}{c} {∥W^{\left(1/2\right)}⊡\left(T-\hat{T}\right)∥}_{F}≲\left(\sum_{k=1}^{n}\sqrt{r_{k}\log\left(d_{k}+\prod_{j\neq k}d_{j}\right)}\mu_{k}\right)\sigma \\ +\left(\sum_{k=1}^{n}r_{k}{∥{\left(W^{\left(-1/2\right)}⊡1_{\Omega }-W^{\left(1/2\right)}\right)}_{\left(k\right)}∥}_{2}\right){∥T∥}_{\infty }, \end{array}$$

*with high probability over the choice of Z, where*

$$\mu_{k}^{2}=\max\left\{\max_{i_{k}}\left(\sum_{i_{1},\cdots ,i_{k-1},i_{k+1},\cdots ,i_{n}}\frac{1_{(i_{1},i_{2},\cdots ,i_{n})\in \Omega }}{W_{i_{1}i_{2}\cdots i_{n}}}\right),\max_{i_{1},\cdots ,i_{k-1},i_{k+1},\cdots ,i_{n}}\left(\sum_{i_{k}}\frac{1_{(i_{1},i_{2},\cdots ,i_{n})\in \Omega }}{W_{i_{1}i_{2}\cdots i_{n}}}\right)\right\}.$$

*and a≲b means that a≤cb for some universal constant c>0.*

**Remark** **2.** *The upper bound in [19] suggests $∥W^{\left(1/2\right)}⊡\left(M-\hat{M}\right){∥}_{F}\leq 2\sqrt{2}r\lambda {∥M∥}_{\infty }$$+4\sqrt{2}\sigma \mu_{1}\sqrt{r\log(d_{1}+d_{2})}$, where $\lambda =∥W^{\left(1/2\right)}-W^{\left(-1/2\right)}\circ 1_{\Omega }∥$ and $\mu_{1}^{2}=max_{(i,j)\in \Omega }\frac{1}{W_{ij}}$, where $\hat{M}$ is obtained by considering the truncated SVD decompositions. Notice that in our result, when n=2, the upper bound becomes $2\sqrt{r\log(d_{1}+d_{2})}\mu \sigma +2r∥W^{\left(1/2\right)}-W^{\left(-1/2\right)}\circ 1_{\Omega }{∥∥M∥}_{\infty }$ with $\mu^{2}=\max\{∥1_{\Omega }\circ W^{\left(-1\right)}{∥}_{\infty },∥1_{\Omega }\circ W^{\left(-1\right)}{∥}_{1}\}$. Since μ in our work is much bigger than the $\mu_{1}$ in [19], the bound in our work is weaker than the one in [19]. The reason is that in order to obtain a general bound for all tensor, the fact that the optimal approximations $\hat{M}$ for a given matrix in the spectral norm and Frobenious norm are the same cannot be applied.
*

#### 3.2.2. Case Study: When Ω∼W

To understand the bounds mentioned above, we also study the case when Ω∼W such that $∥{\left(W^{\left(1/2\right)}-W^{\left(-1/2\right)}⊡1_{\Omega }\right)}_{\left(k\right)}{∥}_{2}$ is small for k=1,⋯,n. Even though the samples are taken randomly in this case, our goal is to understand our upper bounds for deterministic sampling pattern Ω. To make sure that $∥{\left(W^{\left(1/2\right)}-W^{\left(-1/2\right)}⊡1_{\Omega }\right)}_{\left(k\right)}{∥}_{2}$ is small, we need to assume that each entry of W is not too small. For this case, we have the following main results.

**Theorem** **3** (Informal, see Theorems A2 and A7). *Let $W=w_{1}⊗\cdots ⊗w_{n}\in R^{d_{1}\times \cdots \times d_{n}}$ be a CP rank-1 tensor so that for all $\left(i_{1},\cdots ,i_{n}\right)\in \left[d_{1}\right]\times \cdots \times \left[d_{n}\right]$ we have $W_{i_{1}\cdots i_{n}}\in \left[\frac{1}{\sqrt{d_{1}\cdots d_{n}}},1\right]$. Suppose that Ω∼W.*

- ***Upper bound:** Then the following holds with high probability.*
  *For our weighted HOSVD algorithm A, for any Tucker rank-r tensor T with ${∥T∥}_{\infty }\leq \beta$, A returns $\hat{T}=A\left(T_{\Omega }+Z_{\Omega }\right)$ so that with high probability over the choice of Z,*
  $$\begin{array}{cc} \frac{{∥W^{\left(1/2\right)}⊡\left(T-\hat{T}\right)∥}_{F}}{{∥W^{\left(1/2\right)}∥}_{F}}\; & ≲\frac{1}{\sqrt{\left|\Omega \right|}}\left(\beta n^{2}rd^{\frac{n-1}{2}}\log\left(d\right)+\sigma n^{2}r^{1/2}d^{\frac{n-1}{2}}\right), \end{array}$$
  *where $r=\max_{k}\left\{r_{k}\right\}$ and $d=\max_{k}\left\{d_{k}\right\}$.*
- ***Lower bound:** If additionally, W is flat (the entries of W are close), then for our weighted HOSVD algorithm A, there exists some $T\in K_{r}\cap \beta B_{\infty }$ so that with probability at least $\frac{1}{2}$ over the choice of Z,*
  $$\begin{array}{ccc} & & \frac{{∥W^{\left(1/2\right)}⊡\left(A\left(T_{\Omega }+Z_{\Omega }\right)-T\right)∥}_{F}}{{∥W^{\left(1/2\right)}∥}_{F}}\\ & ≳ & \min\left\{\frac{\sigma }{\sqrt{\left|\Omega \right|}}{\left(\frac{\tilde{r}\tilde{d}}{\tilde{d}+2C^{\prime 2}\tilde{r}}\right)}^{\frac{n}{2}},\frac{\sigma }{\sqrt{\left|\Omega \right|}}{\left(\frac{\tilde{r}\tilde{d}}{{\left(\sqrt{\tilde{d}}+\sqrt{2\tilde{r}\log\left(\tilde{r}\right)}C^{\prime }\right)}^{2}}\right)}^{\frac{n}{2}},\frac{\beta }{\sqrt{n\log(\tilde{d})}}\right\}, \end{array}$$
  *where $\tilde{r}=\min_{k}\left\{r_{k}\right\}$, $\tilde{d}=\min_{k}\left\{d_{k}\right\}$, and $C^{\prime }$ is some constant to measure the “flatness" of W.*

**Remark** **3.** *The formal statements in Theorems A2 and A7 are more general than the statements in Theorem 3.*

## 4. Experiments

### 4.1. Simulations for Uniform Sampling Pattern

In this section, we test the performance of our weighted HOSVD algorithm when the sampling pattern arises from uniform random sampling. Consider a tensor T of the form $T=C\times_{1}U_{1}\times_{2}\cdots \times_{n}U_{n}$, where $U_{i}\in R^{d_{i}\times r_{i}}$ and $C\in R^{r_{1}\times \cdots \times r_{n}}$. Let Z be a Gaussian random tensor with $Z_{i_{1}\cdots i_{n}}\in N\left(0,\sigma \right)$ and Ω be the sampling pattern set according to uniform sampling. In this simulation, we compare the results of numerical experiments for using the HOSVD algorithm to solve

(4) $$\hat{T}=\underset{\mathrm{Tucker}\_\mathrm{rank}\;(X)=r}{\mathrm{argmin}}{∥X-Y_{\Omega }∥}_{F},$$

(5) $$\hat{T}=\underset{\mathrm{Tucker}\_\mathrm{rank}\;(X)=r}{\mathrm{argmin}}{∥X-\frac{1}{p}Y_{\Omega }∥}_{F},$$

and

(6) $$\hat{T}=W^{\left(-1/2\right)}⊡\underset{\mathrm{Tucker}\_\mathrm{rank}\;(X)=r}{\mathrm{argmin}}{∥X-W^{\left(-1/2\right)}⊡Y_{\Omega }∥}_{F},$$

where $p=\frac{\left|\Omega \right|}{\prod_{k=1}^{n}d_{k}}$ and $Y_{\Omega }=T_{\Omega }+Z_{\Omega }$.

**Figure 1:**
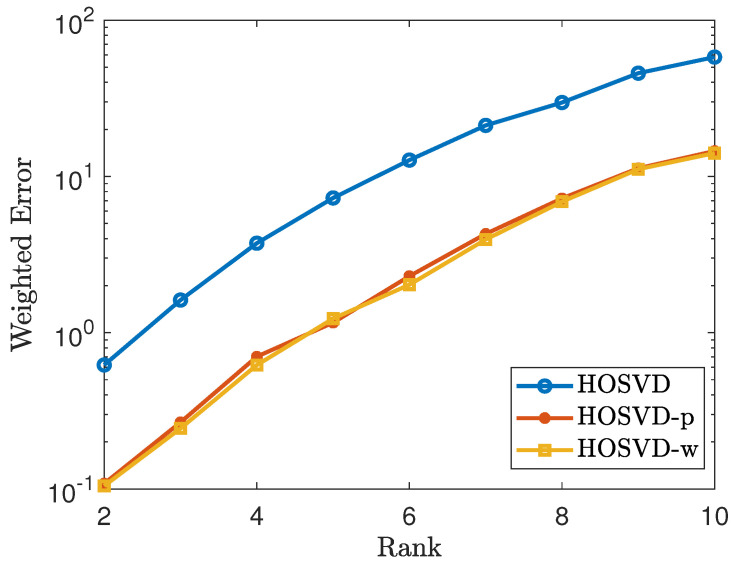
Tensor of size 100×100×100 using the uniform sampling pattern: plots the errors of the form $\frac{∥W^{\left(1/2\right)}⊡\left(\hat{T}-T\right){∥}_{F}}{∥W^{\left(1/2\right)}{∥}_{F}}$. The lines labeled as HOSVD, HOSVD-p and HOSVD-w represent the results for solving (4), (5) and (6), respectively.

**Figure 2:**
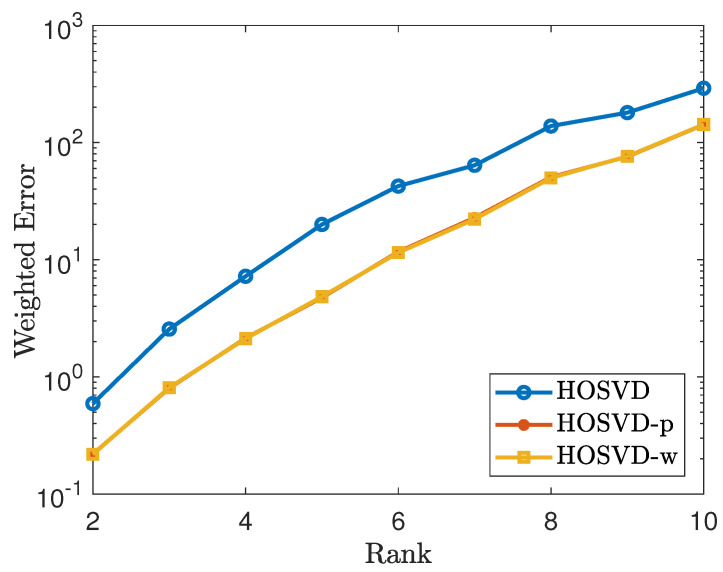
Tensor of size 50×50×30×30 using the uniform sampling pattern: plots the errors of the form $\frac{∥W^{\left(1/2\right)}⊡\left(\hat{T}-T\right){∥}_{F}}{∥W^{\left(1/2\right)}{∥}_{F}}$. The lines labeled as HOSVD, HOSVD-p and HOSVD-w represent the results for solving (4), (5) and (6), respectively.

First, we generate a synthetic sampling set Ω with sampling rate SR$:\;=\frac{\left|\Omega \right|}{\prod_{k=1}^{n}d_{k}}=30\%$ and find a weight tensor W by solving

(7) $$W=\underset{X≻0,\mathrm{rank}(X)=1}{\mathrm{argmin}}{∥X-1_{\Omega }∥}_{F}$$

via the alternating least squares method for the non-negative CP decomposition. Next, we generate synthetic tensors $T\in R^{d_{1}\times \cdots \times d_{n}}$ of the form $C\times_{1}U_{1}\times_{2}\cdots \times_{n}U_{n}$ with n=3,4 with $\mathrm{rank}\;(T_{\left(i\right)})=r$, where i=1,⋯,n, and *r* varies from 2 to 10. Then we add mean zero Gaussion random noise Z with variance $\sigma =10^{-2}$ so that a new tensor is generated, which is denoted by Y=T+Z. Then we solve the tensor completion problems (4), (5) and (6) by the HOSVD procedure. For each fixed low-rank tensor, we average over 20 tests. We measure error using the weighted Frobenius norm. The simulation results are reported in Figure 1 and Figure 2. Figure 1 shows the results for the tensor of size 100×100×100 and Figure 2 shows the results for the tensor of size 50×50×30×30, where the weighted error is of the form $\frac{∥W^{\left(1/2\right)}⊡\left(\hat{T}-T\right){∥}_{F}}{∥W^{\left(1/2\right)}∥}$. These figures demonstrate that using our weighted samples performs more efficiently than using the original samples. For the uniform sampling case, the *p* weighted samples and W weighted samples exhibit similar performance.

### 4.2. Simulation for Non-Uniform Sampling Pattern

**Figure 3:**
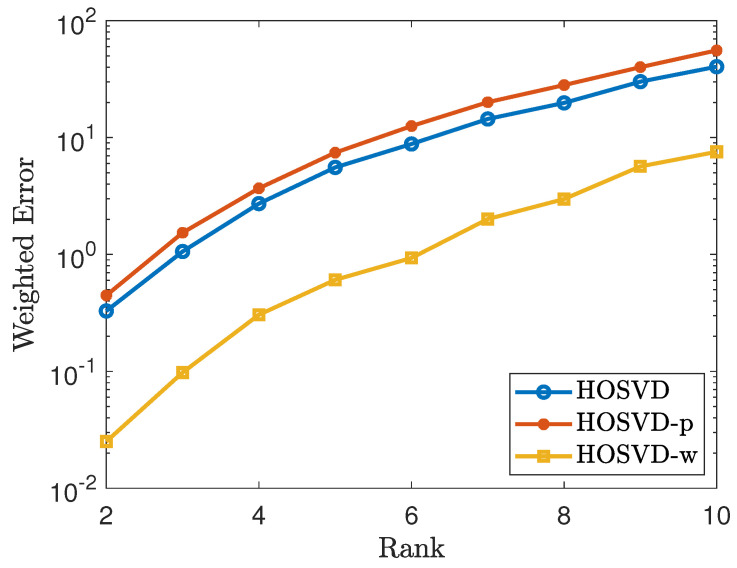
Tensor of size 100×100×100 using the non-uniform sampling pattern: plots the errors of the form $\frac{∥W^{\left(1/2\right)}⊡\left(\hat{T}-T\right){∥}_{F}}{∥W^{\left(1/2\right)}{∥}_{F}}$. The lines labeled as HOSVD, HOSVD-p and HOSVD-w represent the results for solving (4), (5) and (6), respectively.

**Figure 4:**
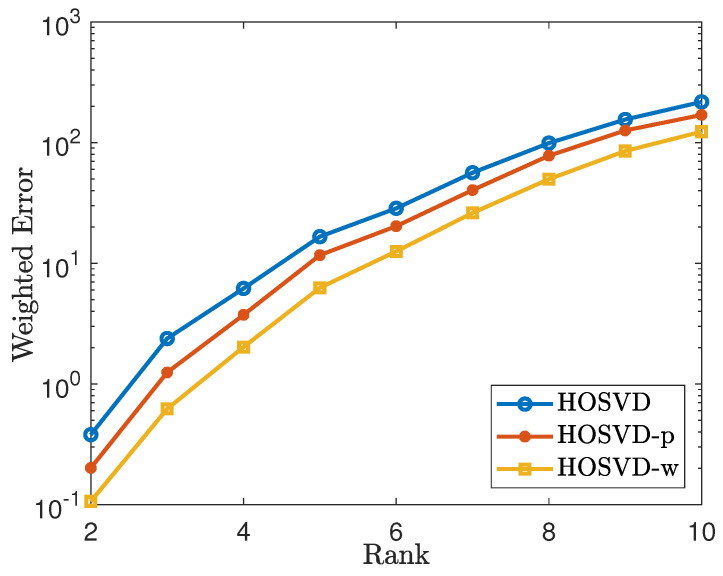
Tensor of size 50×50×30×30 using the non-uniform sampling pattern: plots the errors of the form $\frac{∥W^{\left(1/2\right)}⊡\left(\hat{T}-T\right){∥}_{F}}{∥W^{\left(1/2\right)}{∥}_{F}}$. The lines labeled as HOSVD, HOSVD-p and HOSVD-w represent the results for solving (4), (5) and (6), respectively.

To generate a non-uniform sampling pattern with sampling rate 30%, we first generate a CP rank 1 tensor of the form $H=〚1;h_{1},\cdots ,h_{n}〛$, where $h_{i}=\left(u_{i}1_{\left\lceil d_{i}/2\right\rceil },v_{i}1_{\left\lfloor d_{i}/2\right\rfloor }\right)$$0<u_{i},v_{i}\leq 1$. Let Ω∼H. Then we repeat the process as in Section 4.1. The simulation results are shown in Figure 3 and Figure 4. As shown in figures, the results using our proposed weighted samples perform more efficiently than using the *p* weighted samples.

**Figure 5:**
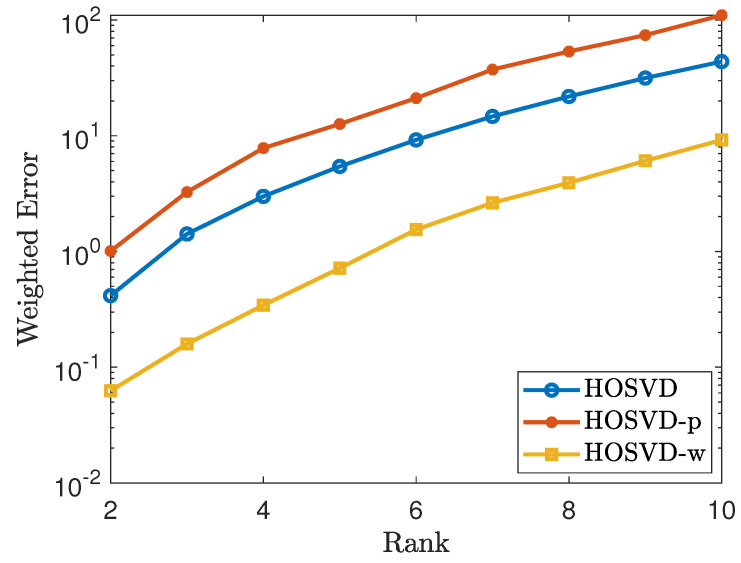
Tensor of size 100×100×100 using the non-uniform sampling pattern and with the SV-rank as the input rank: plots the errors of the form $\frac{∥W^{\left(1/2\right)}⊡\left(\hat{T}-T\right){∥}_{F}}{∥W^{\left(1/2\right)}{∥}_{F}}$.

**Remark** **4.** *When we use the HOSVD procedure to solve (4), (5), and (6), we need (an estimate of) the Tucker rank as input. Instead of inputting the real rank of the true tensor, we could also use the rank that is estimated by considering the decay of the singular values for the unfolded matrices of the sampled tensor $Y_{\Omega }$ as the input rank, which we call SV-rank. The simulation results for the non-uniform sampling pattern with SV-rank as input are reported in Figure 5. The simulation shows that the weighted HOSVD algorithm performs more efficiently than using the p weighted samples or the original samples. Comparing Figure 5 with Figure 3, we could observe that using the estimated rank as input for HOSVD procedure performs even better than using the real rank as input. This observation motivates a way to find a “good" rank as input for HOSVD procedure.*

**Remark** **5.** *We only provide guarantees on the performance in the weighted Frobenius norm, (as we report the weighted error $\frac{∥W^{\left(1/2\right)}⊡\left(\hat{T}-T\right){∥}_{F}}{∥W^{\left(1/2\right)}{∥}_{F}}$), our procedures exhibit good empirical performance even in the usual relative error $\frac{∥\hat{T}{-T∥}_{F}}{{∥T∥}_{F}}$ when the Tucker rank of the tensor is relatively low. However, we observe that the advantages of weighted HOSVD scheme tend to be diminished in terms of relative error when the Tucker rank increases. This result is not surprising since the entries are treated unequally in scheme (6). Therefore we leave the investigation on relative error and the tensor rank for future work.*

### 4.3. Test for Real Data

**Figure 6:**
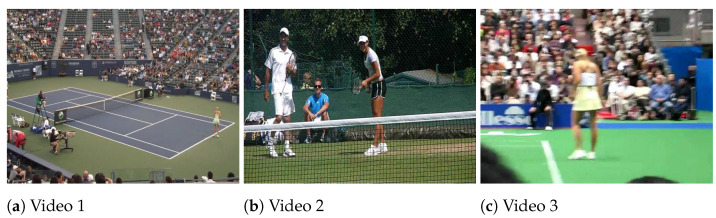
The first frame of videos [50].

In this section, we test our weighted HOSVD algorithm for tensor completion on three videos, see [50]. The dataset is the tennis-serve data from an Olympic Sports Dataset [51]. One can download the dataset from http://vision.stanford.edu/Datasets (accessed date 10 May 2021). There are a lot of videos in the zip file and we only choose three of them: “d2P_zx_JeoQ_00120_00515.seq” (video 1), “gs3sPDfbeg4_00082_00229.seq”(video 2), and “VADoc-AsyXk_00061_ 0019.seq” (video 3). The three videos are color video. In our simulation, we use the same setup as the one in [50], and choose 30 frames evenly from each video. For each frame, the size is scaled to 360×480×3, so each video is transformed into a 4-D tensor data of size 360×480×3×30. The first frame of each video after preprocessing is illustrated in Figure 6.

We implement the experiments for different sampling rates of 10%, 30%, 50%, and 80% to generate uniform and non-uniform sampling patterns Ω. In our implementation, we use the SV-rank of $T_{\Omega }$ as the input rank. According to the generated sampling pattern, we find a weight tensor W and find estimates ${\hat{T}}_{1}$ and ${\hat{T}}_{2}$ by considering (4) and (6) respectively, using the input Tucker rank r. The entries on $T_{1}$ and $T_{2}$ are forced to be the observed data. The Signal to Noise Ratio (SNR)

$$SNR\left(\hat{T}\right)=-20\log_{10}\left(\frac{∥\hat{T}{-T∥}_{F}}{{∥T∥}_{F}}\right)$$

are computed and the simulation results are reported in Table 1 and Table 2. As shown in the tables, applying HOSVD process to (6) can give a better result than applying HOSVD process to (4) directly regardless of the uniformity of the sampling pattern.

**Figure 7:**
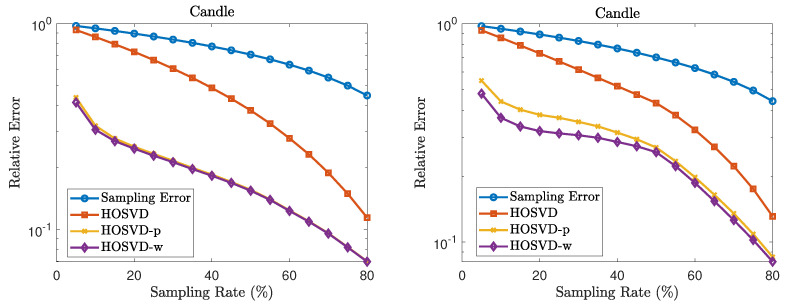
Relation between relative error and sampling rate for the dataset “candle_4_A” using [5,5,5] as the input rank for HOSVD process. The left figure records the relative error for the uniform sampling pattern and the right figure for the non-uniform sampling pattern. The sampling error stands for the relative error between the original video and the video with masked entries estimated to be zeros, hence should approximately equal to $\sqrt{1-SR}$, where SR is the sampling rate.

Finally, we test the proposed weighted HOSVD algorithm on real candle video data named “candle_4_A” [52] (The dataset can be downloaded from the Dynamic Texture Toolbox in http://www.vision.jhu.edu/code/ (accessed date 10 May 2021). We have tested the relation between the relative errors and the sampling rates using r=(5,5,5) as the input rank for HOSVD algorithm. The relative errors are presented in Figure 7. The simulation results also show that the proposed weighted HOSVD algorithm can implement tensor completion efficiently.

### 4.4. The Application of Weighted HOSVD on Total Variation Minimization

As shown in the previous simulations, the weighted HOSVD decomposition can provide better results for tensor completion by comparing with HOSVD. There are a bunch of algorithms that are Sensitive to initialization. Additionally, real applications may have higher requirements for accuracy. Therefore, it is meaningful to combine our weighted HOSVD with other algorithms in order to further improve the performance. In this section, we would consider the application of weighted HOSVD decomposition on the total variation minimization algorithm. As a traditional approach, the total variation minimization (TVM), is broadly applied in studies about image recovery and denoising. While the earliest research could trace back to 1992 [53]. The later studies combined TVM and other low rank approximation algorithms such as Nuclear Norm Minimization (see e.g., [54,55,56]) and HOSVD (e.g., [57,58,59]) in order to achieve better performance in image and video completion tasks.

Motivated by the matrix TV minimization, we proposed the tensor TV minimization which is summarized in Algorithm 1. In Algorithm 1, the Laplacian operator computes the divergence of all-dimension gradients for each entry of the tensor. The shrink operator simply moves the input towards 0 with distance λ, or formally defined as:

shrink(x,λ)=sign(x)·max(|x|−λ,0)

For the initialization of $X^{0}$ in Algorithm 1, we assign $X^{0}$ to be the output of the result from HOSVD-w.

**Figure 8:**
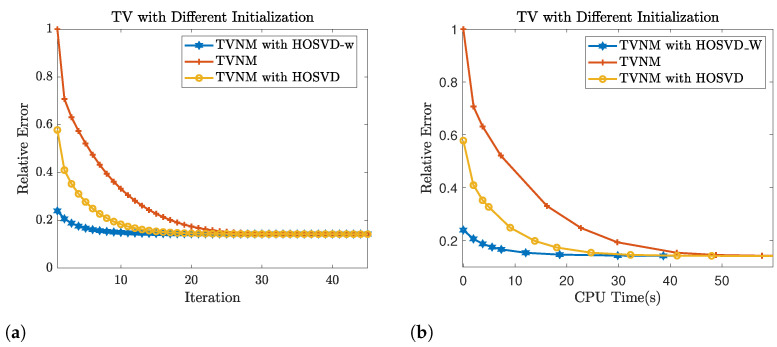
Convergence comparison between total variation minimization (TVM) with HOSVD-w, 0, and HOSVD as initialization on video 1 with SR = 50%: (**a**) the relative error $\frac{∥\hat{T}{-T∥}_{F}}{{∥T∥}_{F}}$ vs. number of iterations. (**b**) the relative error v.s. total computational CPU time(initialization + completion).

Applying the same experiment setting as in Section 4.3, we evaluate the performance of the cocktail approach as well as the regular HOSVD approach. We report the simulation results in Table 1 and we measure the performances by considering the signal to noise ratio(SNR). As shown in Table 1, the total variation minimization could be applied to further improve the result of (6). Specifically, the TVM with 0 as initialization performs similar to TVM with HOSVD-w as initialization when the observed rate is high, but the HOSVD-w initialization could improve the performance of TVM when the observed rate is very low (e.g., 10%). Additionally, we compared the decay of relative error for using the weighted HOSVD output as initialization and the default initialization ($X^{0}=0$). The iterative results are shown in Figure 8, and it shows that using the result from weighted HOSVD as an initialization could notably reduce the iterations of TV-minimization for achieving the convergence threshold ($∥X^{k}-X^{k-1}{∥}_{F}<10^{-4}$).
**Algorithm 1:** TV Minimization for Tensor.
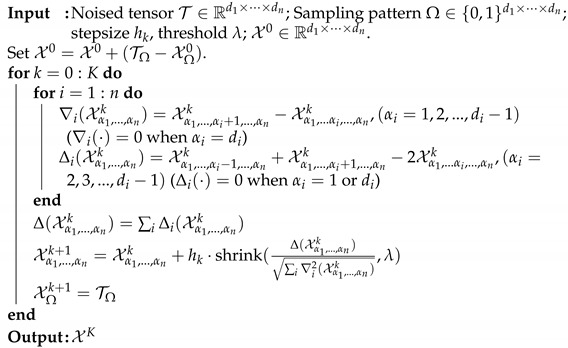


## 5. Conclusions

In this paper, we propose a simple but efficient algorithm named the weighted HOSVD algorithm for recovering an underlying low-rank tensor from noisy observations. For this algorithm, we provide upper and lower error bounds that measure the difference between the estimates and the true underlying low-rank tensor. The efficiency of our proposed weighted HOSVD algorithm is also shown by numerical simulations. Additionally, the result of our weighted HOSVD algorithm can be used as an initialization for the total variation minimization algorithm, which shows that using our method as an initialization for the total variation minimization algorithm can increasingly reduce the iterative steps leading to improved overall performance in reconstruction (see our conference paper [60]). It would be interesting for future work to combine the weighted HOSVD algorithm with other algorithms to achieve more accurate results for tensor completion in many settings.

## Figures and Tables

**Table 1:** Signal to noise ratio (SNR) and elapsed time (in second) for Higher Order Singular Value Decomposition (HOSVD) and HOSVD-w on video data with uniform sampling pattern. The HOSVD-w and HOSVD-p behave very similar for uniform sampling hence we integrate the results into one column.

Video	SR	Input Rank	HOSVD-w+TV	HOSVD	HOSVD-w/HOSVD-p	TVM
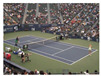	10%	[71735]	13.29 (16.3 s)	1.27 (3.74 s)	10.15 (11.4 s)	13.04 (41.3 s)
	30%	[181036]	16.96 (14.0 s)	4.26 (4.01 s)	12.05 (7.23 s)	17.05 (29.7 s)
	50%	[264311]	19.60 (12.2 s)	8.21 (2.99 s)	14.59 (7.03 s)	19.68 (23.8 s)
	80%	[4747322]	24.90 (11.5 s)	17.29 (6.55 s)	19.75 (8.08 s)	25.01 (18.1 s)
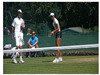	10%	[28637]	10.98 (13.1 s)	1.19 (4.20 s)	7.88 (8.76 s)	10.89 (42.2 s)
	30%	[3418315]	14.44 (16.1 s)	4.11 (3.80 s)	10.40 (7.51 s)	14.50 (31.4 s)
	50%	[353339]	16.95 (15.3 s)	7.85 (5.86 s)	12.84 (7.64 s)	16.96 (26.6 s)
	80%	[5650321]	22.21 (15.1 s)	16.51 (7.24 s)	18.64 (8.45 s)	22.19 (18.4 s)
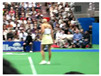	10%	[129310]	12.34 (16.1 s)	1.22 (2.73 s)	8.46 (9.88 s)	12.23 (45.7 s)
	30%	[2024311]	17.10 (15.3 s)	4.24 (3.17 s)	11.62 (7.62 s)	17.19 (35.3 s)
	50%	[2532314]	20.44 (12.3 s)	8.20 (3.92 s)	14.54 (5.85 s)	20.49 (28.9 s)
	80%	[5072330]	26.80 (12.4 s)	18.03 (8.40 s)	21.38 (8.93s)	26.71 (20.9 s)

**Table 2:** Signal to noise ratio (SNR) for HOSVD and HOSVD-w on video data with non-uniform sampling pattern.

Video	SR	Input Rank	HOSVD	HOSVD-w	HOSVD-p
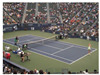	10%	$\left[6\;13\;3\;3\right]$	1.09	10.07	5.56
	30%	$\left[10\;28\;3\;16\right]$	3.74	11.81	7.53
	50%	$\left[21\;41\;3\;14\right]$	7.05	13.22	10.73
	80%	$\left[44\;57\;3\;26\right]$	15.76	19.60	17.39
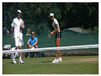	10%	$\left[38\;11\;3\;2\right]$	1.13	8.04	4.33
	30%	$\left[26\;19\;3\;16\right]$	3.79	10.13	6.80
	50%	$\left[30\;27\;3\;10\right]$	7.15	12.57	10.14
	80%	$\left[53\;50\;3\;23\right]$	14.81	18.55	16.31
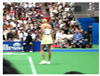	10%	$\left[16\;11\;3\;2\right]$	1.09	8.31	4.73
	30%	$\left[17\;23\;3\;17\right]$	3.76	11.05	6.87
	50%	$\left[24\;38\;3\;14\right]$	7.18	13.78	9.99
	80%	$\left[47\;69\;3\;22\right]$	15.88	20.82	16.02

## Data Availability

In this work, the following the pre-existing reference databases have been used for our evaluations: tennis-seve dataset ([50,51]) and candel video data ([52]).They are publicly available at: http://vision.stanford.edu/Datasets/OlympicSports/ (accessed date 10 May 2021); http://www.vision.jhu.edu/code/ (accessed date 10 May 2021).

## Appendix A. Proof for Theorem 1

In this appendix, we provide the proof for Theorem 1.

**Proof of Theorem** Let $Y_{\Omega }=T_{\Omega }+Z_{\Omega }$.

$$\begin{array}{ccc} & & {∥W^{\left(1/2\right)}⊡\left(T-\hat{T}\right)∥}_{F}\\ & = & {∥W^{\left(1/2\right)}⊡T-W^{\left(-1/2\right)}⊡Y_{\Omega }+W^{\left(-1/2\right)}⊡Y_{\Omega }-W^{\left(1/2\right)}⊡\hat{T}∥}_{F}\\ & \leq & {∥W^{\left(1/2\right)}⊡T-W^{\left(-1/2\right)}⊡Y_{\Omega }∥}_{F}+{∥W^{\left(-1/2\right)}⊡Y_{\Omega }-W^{\left(1/2\right)}⊡\hat{T}∥}_{F}\\ & \leq & 2{∥W^{\left(1/2\right)}⊡T-W^{\left(-1/2\right)}⊡Y_{\Omega }∥}_{F}\\ & = & 2{∥W^{\left(1/2\right)}⊡T-W^{\left(-1/2\right)}⊡\left(T_{\Omega }+Z_{\Omega }\right)∥}_{F}\\ & \leq & 2{∥W^{\left(1/2\right)}⊡T-W^{\left(-1/2\right)}⊡1_{\Omega }⊡T∥}_{F}+2{∥W^{\left(-1/2\right)}⊡Z_{\Omega }∥}_{F}\\ & \leq & 2{∥T⊡(W^{\left(1/2\right)}-W^{\left(-1/2\right)}⊡1_{\Omega })∥}_{F}+2{∥W^{\left(-1/2\right)}⊡Z_{\Omega }∥}_{F}\\ & \leq & 2{∥T∥}_{\infty }{∥W^{\left(1/2\right)}-W^{\left(-1/2\right)}⊡1_{\Omega }∥}_{F}+2{∥W^{\left(-1/2\right)}⊡Z_{\Omega }∥}_{F}. \end{array}$$

Thus, we have that

(A1) $${∥W^{\left(1/2\right)}⊡\left(T-\hat{T}\right)∥}_{F}\leq 2{∥T∥}_{\infty }{∥W^{\left(1/2\right)}-W^{\left(-1/2\right)}⊡1_{\Omega }∥}_{F}+2{∥W^{\left(-1/2\right)}⊡Z_{\Omega }∥}_{F}.$$

Next, let’s estimate ${∥W^{\left(-1/2\right)}⊡Z_{\Omega }∥}_{F}$. Notice that

$${∥W^{-(1/2)}⊡Z_{\Omega }∥}_{F}^{2}=\sum_{(i_{1},\cdots ,i_{n})\in \Omega }\frac{Z_{i_{1}\cdots i_{n}}^{2}}{W_{i_{1}\cdots i_{n}}}$$

$$\begin{array}{ccc} P\left\{{∥W^{\left(-1/2\right)}⊡Z_{\Omega }∥}_{F}\geq t\right\} & = & P\left\{e^{s{∥W^{\left(-1/2\right)}⊡Z_{\Omega }∥}_{F}^{2}}\geq e^{st^{2}}\right\}\\ & \leq & e^{-st^{2}}E\left(\exp\left(s{∥W^{\left(-1/2\right)}⊡Z_{\Omega }∥}_{F}^{2}\right)\right)\\ & \leq & e^{-st^{2}}\prod_{(i_{1},\cdots ,i_{n})\in \Omega }E\left(\exp\left(\frac{sZ_{i_{1}\cdots i_{n}}^{2}}{W_{i_{1}\cdots i_{n}}}\right)\right)\\ & = & e^{-st^{2}}\prod_{(i_{1},\cdots ,i_{n})\in \Omega }\left(\frac{1}{\sqrt{1-2\sigma^{2}s/W_{i_{1}\cdots i_{n}}}}\right) \end{array}$$

Recall that $\mu^{2}=\max_{(i_{1},\cdots ,i_{n})\in \Omega }\frac{1}{W_{i_{1},\cdots ,i_{n}}}$. By choosing $s=\frac{1}{4\sigma^{2}\mu^{2}}$, we have that

$$\begin{array}{c} P\left\{{∥W^{-(1/2)}\circ Z_{\Omega }∥}_{F}\geq t\right\}\leq \exp\left(-\frac{t^{2}}{4\sigma^{2}\mu^{2}}\right)2^{|\Omega |/2}. \end{array}$$

We conclude that with probability at least $1-2^{-|\Omega |/2}$,

$${∥W^{\left(-1/2\right)}\circ Z_{\Omega }∥}_{F}\leq 2\sigma \mu \sqrt{\left|\Omega |\log(2\right)}.$$

Plugging this into (A1) proves the theorem. □

## Appendix B. Proof of Theorems 2 and 3

In this appendix, we provide the proofs for the results related with the weighted HOSVD algorithm. The general upper bound for weighted HOSVD in Theorem 2 is restated in Appendix B.1 and its proof is also presented there. If the sampling pattern Ω is generated according to the weight tensor W, the related results in Theorem 3 are illustrated in Appendix B.2.

### Appendix B.1. General Upper Bound for Weighted HOSVD Algorithm

**Theorem** **A1.**
*Let $W=w_{1}⊗\cdots ⊗w_{n}\in R^{d_{1}\times \cdots \times d_{n}}$ have strictly positive entries, and fix $\Omega \subseteq \left[d_{1}\right]\times \cdots \times \left[d_{n}\right]$. Suppose that $T\in R^{d_{1}\times \cdots \times d_{n}}$ has Tucker rank $r=[r_{1}\;\cdots \;r_{n}]$. Suppose that $Z_{i_{1}\cdots i_{n}}∼N\left(0,\sigma^{2}\right)$ and let*

$$\hat{T}=W^{\left(-1/2\right)}⊡\left(\left(W^{\left(-1/2\right)}⊡Y_{\Omega }\right)\times_{1}{\hat{U}}_{1}{\hat{U}}_{1}^{T}\times_{2}\cdots \times_{n}{\hat{U}}_{n}{\hat{U}}_{n}^{T}\right)$$

*where ${\hat{U}}_{1},\cdots ,{\hat{U}}_{n}$ are obtained by HOSVD approximation process, where $Y_{\Omega }=1_{\Omega }⊡\left(T+Z\right)$. Then with probability at least $1-\sum_{i=1}^{n}\frac{1}{d_{i}+\prod_{j\neq i}d_{j}}$ over the choice of Z,*

$$\begin{array}{cc} \; & {∥W^{\left(1/2\right)}⊡\left(T-\hat{T}\right)∥}_{F}\\ \leq & \left(\sum_{k=1}^{n}6\sqrt{r_{k}\log\left(d_{k}+\prod_{j\neq k}d_{j}\right)}\mu_{k}\right)\sigma +\left(\sum_{k=1}^{n}3r_{k}{∥{\left(W^{\left(-1/2\right)}⊡1_{\Omega }-W^{\left(1/2\right)}\right)}_{\left(k\right)}∥}_{2}\right){∥T∥}_{\infty }. \end{array}$$

*where*

$$\mu_{k}^{2}=\max\left\{\max_{i_{k}}\left(\sum_{i_{1},\cdots ,i_{k-1},i_{k+1},\cdots ,i_{n}}\frac{1_{(i_{1},i_{2},\cdots ,i_{n})\in \Omega }}{W_{i_{1}i_{2}\cdots i_{n}}}\right),\max_{i_{1},\cdots ,i_{k-1},i_{k+1},\cdots ,i_{n}}\left(\sum_{i_{k}}\frac{1_{(i_{1},i_{2},\cdots ,i_{n})\in \Omega }}{W_{i_{1}i_{2}\cdots i_{n}}}\right)\right\}.$$

**Proof.** Recall that $T_{\Omega }=1_{\Omega }⊡T$ and $Z_{\Omega }=1_{\Omega }⊡Z$. First we have the following estimations.

$$\begin{array}{ccc} & & {∥W^{\left(1/2\right)}⊡\left(\hat{T}-T\right)∥}_{F}\\ & = & {∥\left(W^{\left(-1/2\right)}⊡Y_{\Omega }\right)\times_{1}{\hat{U}}_{1}{\hat{U}}_{1}^{T}\times_{2}\cdots \times_{n}{\hat{U}}_{n}{\hat{U}}_{n}^{T}-\left(W^{\left(1/2\right)}⊡T\right)\times_{1}U_{1}U_{1}^{T}\times_{2}\cdots \times_{n}U_{n}U_{n}^{T}∥}_{F}\\ & \leq & {∥\left(\left(W^{\left(-1/2\right)}⊡Y_{\Omega }\right)\times_{1}{\hat{U}}_{1}{\hat{U}}_{1}^{T}-\left(W^{\left(1/2\right)}⊡T\right)\times_{1}U_{1}U_{1}^{T}\right)\times_{2}{\hat{U}}_{2}{\hat{U}}_{2}^{T}\times_{3}\cdots \times_{n}{\hat{U}}_{n}{\hat{U}}_{n}^{T}∥}_{F}\\ & & +{∥\left(W^{\left(1/2\right)}⊡T\right)\left(\times_{2}U_{2}U_{2}^{T}\times_{3}\cdots \times_{n}U_{n}U_{n}^{T}-\times_{2}{\hat{U}}_{2}{\hat{U}}_{2}^{T}\times_{3}\cdots \times_{n}{\hat{U}}_{n}{\hat{U}}_{n}^{T}\right)∥}_{F}\\ & \leq & \sqrt{2r_{1}}{∥{\hat{U}}_{1}{\hat{U}}_{1}^{T}{\left(W^{\left(-1/2\right)}⊡Y_{\Omega }\right)}_{\left(1\right)}-U_{1}U_{1}^{T}{\left(W^{\left(1/2\right)}⊡T\right)}_{\left(1\right)}∥}_{2}+\sum_{k=2}^{n}∥(W^{\left(1/2\right)}⊡T)\\ & & {\times_{2}{\hat{U}}_{2}{\hat{U}}_{2}^{T}\times_{3}\cdots \times_{k-1}{\hat{U}}_{k-1}{\hat{U}}_{k-1}^{T}\times_{k}\left(U_{k}U_{k}^{T}-{\hat{U}}_{k}{\hat{U}}_{k}^{T}\right)\times_{k+1}U_{k+1}U_{k+1}^{T}\times_{k+2}\cdots \times_{n}U_{n}U_{n}^{T}∥}_{F}\\ & \leq & \sqrt{2r_{1}}{∥{\hat{U}}_{1}{\hat{U}}_{1}^{T}{\left(W^{\left(-1/2\right)}⊡Y_{\Omega }\right)}_{\left(1\right)}-{\left(W^{\left(1/2\right)}⊡T\right)}_{\left(1\right)}∥}_{2}+\sum_{k=2}^{n}\sqrt{r_{k}}{∥\left(U_{k}U_{k}^{T}-{\hat{U}}_{k}{\hat{U}}_{k}^{T}\right){\left(W^{\left(1/2\right)}⊡T\right)}_{\left(k\right)}∥}_{2}\\ & \leq & \sqrt{2r_{1}}\left({∥{\hat{U}}_{1}{\hat{U}}_{1}^{T}{\left(W^{\left(-1/2\right)}⊡Y_{\Omega }\right)}_{\left(1\right)}-{\left(W^{\left(-1/2\right)}⊡Y_{\Omega }\right)}_{\left(1\right)}∥}_{2}+{∥{\left(W^{\left(-1/2\right)}⊡Y_{\Omega }\right)}_{\left(1\right)}-{\left(W^{\left(1/2\right)}⊡T\right)}_{\left(1\right)}∥}_{2}\right)\\ & & +\sum_{k=2}^{n}\sqrt{r_{k}}{∥\left(U_{k}U_{k}^{T}-{\hat{U}}_{k}{\hat{U}}_{k}^{T}\right){\left(W^{\left(1/2\right)}⊡T\right)}_{\left(k\right)}∥}_{2}\\ & \leq & 2\sqrt{2r_{1}}{∥{\left(W^{\left(-1/2\right)}⊡Y_{\Omega }\right)}_{\left(1\right)}-{\left(W^{\left(1/2\right)}⊡T\right)}_{\left(1\right)}∥}_{2}+\sum_{k=2}^{n}\sqrt{r_{k}}{∥\left(U_{k}U_{k}^{T}-{\hat{U}}_{k}{\hat{U}}_{k}^{T}\right){\left(W^{\left(1/2\right)}⊡T\right)}_{\left(k\right)}∥}_{2}. \end{array}$$

Notice that

$$\begin{array}{ccc} & & {∥\left(U_{k}U_{k}^{T}-{\hat{U}}_{k}{\hat{U}}_{k}^{T}\right){\left(W^{\left(1/2\right)}⊡T\right)}_{\left(k\right)}∥}_{2}\\ & = & {∥{\left(W^{\left(1/2\right)}⊡T\right)}_{\left(k\right)}-{\hat{U}}_{k}{\hat{U}}_{k}^{T}{\left(W^{\left(1/2\right)}⊡T\right)}_{\left(k\right)}∥}_{2}\\ & \leq & {∥{\left(W^{\left(1/2\right)}⊡T\right)}_{\left(k\right)}-{\left(W^{\left(-1/2\right)}⊡Y_{\Omega }\right)}_{\left(k\right)}∥}_{2}+{∥{\hat{U}}_{k}{\hat{U}}_{k}^{T}{\left(W^{\left(1/2\right)}⊡T-W^{\left(-1/2\right)}⊡Y_{\Omega }\right)}_{\left(k\right)}∥}_{2}+\\ & & {∥{\left(W^{\left(-1/2\right)}⊡Y_{\Omega }\right)}_{\left(k\right)}-{\hat{U}}_{k}{\hat{U}}_{k}^{T}{\left(W^{\left(-1/2\right)}⊡Y_{\Omega }\right)}_{\left(k\right)}∥}_{2}\\ & \leq & 3{∥{\left(W^{\left(1/2\right)}⊡T\right)}_{\left(k\right)}-{\left(W^{\left(-1/2\right)}⊡Y_{\Omega }\right)}_{\left(k\right)}∥}_{2}. \end{array}$$

Therefore, we have

(A2) $$\begin{array}{ccc} & & {∥W^{\left(1/2\right)}⊡\left(\hat{T}-T\right)∥}_{F}\leq \sum_{k=1}^{n}3\sqrt{r_{k}}{∥{\left(W^{\left(1/2\right)}⊡T\right)}_{\left(k\right)}-{\left(W^{\left(-1/2\right)}⊡Y_{\Omega }\right)}_{\left(k\right)}∥}_{2}. \end{array}$$

Next, to estimate ${∥{\left(W^{\left(-1/2\right)}⊡Y_{\Omega }-W^{\left(1/2\right)}⊡T\right)}_{\left(k\right)}∥}_{2}$ for k=1,⋯,n. Let us consider the case when k=1. Other cases can be derived similarly. Using the fact that $T_{\left(1\right)}$ has rank $r_{1}$ and ${∥T_{\left(1\right)}∥}_{\max}\leq \sqrt{r_{1}}{∥T_{\left(1\right)}∥}_{\infty }=\sqrt{r_{1}}{∥T∥}_{\infty }$, we conclude that

$$\begin{array}{ccc} & & {∥{\left(W^{\left(-1/2\right)}⊡Y_{\Omega }-W^{\left(1/2\right)}⊡T\right)}_{\left(1\right)}∥}_{2}\\ & = & {∥{\left(W^{\left(-1/2\right)}⊡T_{\Omega }-W^{\left(1/2\right)}⊡T\right)}_{\left(1\right)}+{\left(W^{\left(-1/2\right)}⊡Z_{\Omega }\right)}_{\left(1\right)}∥}_{2}\\ & \leq & {∥{\left(W^{\left(-1/2\right)}⊡T_{\Omega }-W^{\left(1/2\right)}⊡T\right)}_{\left(1\right)}∥}_{2}+{∥{\left(W^{\left(-1/2\right)}⊡Z_{\Omega }\right)}_{\left(1\right)}∥}_{2}\\ & = & {∥{\left(W^{\left(-1/2\right)}⊡1_{\Omega }-W^{\left(1/2\right)}\right)}_{\left(1\right)}⊡T_{\left(1\right)}∥}_{2}+{∥{\left(W^{\left(-1/2\right)}⊡Z_{\Omega }\right)}_{\left(1\right)}∥}_{2}\\ & \leq & {∥T_{\left(1\right)}∥}_{\max}{∥{\left(W^{\left(-1/2\right)}⊡1_{\Omega }-W^{\left(1/2\right)}\right)}_{\left(1\right)}∥}_{2}+{∥{\left(W^{\left(-1/2\right)}⊡Z_{\Omega }\right)}_{\left(1\right)}∥}_{2}\\ & \leq & \sqrt{r_{1}}{∥T∥}_{\infty }{∥{\left(W^{\left(-1/2\right)}⊡1_{\Omega }-W^{\left(1/2\right)}\right)}_{\left(1\right)}∥}_{2}+{∥{\left(W^{\left(-1/2\right)}⊡Z_{\Omega }\right)}_{\left(1\right)}∥}_{2}. \end{array}$$

To bound ${∥{\left(W^{\left(-1/2\right)}⊡Z_{\Omega }\right)}_{\left(1\right)}∥}_{2}$, we consider

$${\left(W^{\left(-1/2\right)}⊡Z_{\Omega }\right)}_{\left(1\right)}=\sum_{i_{1},\cdots ,i_{n}}\frac{1_{(i_{1},\cdots ,i_{n})\in \Omega }Z_{i_{1}\cdots i_{n}}}{\sqrt{W_{i_{1}\cdots i_{n}}}}e_{i_{1}}{\left(e_{i_{2}}⊗\cdots ⊗e_{i_{n}}\right)}^{T},$$

where $e_{i_{k}}$ is the $i_{k}$-th standard basis vector of $R^{d_{k}}$. Please note that

$$\begin{array}{ccc} & & \sum_{i_{1},\cdots ,i_{n}}\frac{1_{(i_{1},\cdots ,i_{n})\in \Omega }}{W_{i_{1}\cdots i_{n}}}e_{i_{1}}{\left(e_{i_{2}}⊗\cdots ⊗e_{i_{n}}\right)}^{T}\left(e_{i_{2}}⊗\cdots ⊗e_{i_{n}}\right)e_{i_{1}}^{T}\\ & = & \sum_{i_{1},\cdots ,i_{n}}\frac{1_{(i_{1},\cdots ,i_{n})\in \Omega }}{W_{i_{1}\cdots i_{n}}}e_{i_{1}}e_{i_{1}}^{T}. \end{array}$$

Therefore,

$$\begin{array}{ccc} & & {∥\sum_{i_{1},\cdots ,i_{n}}\frac{1_{(i_{1},\cdots ,i_{n})\in \Omega }}{W_{i_{1}\cdots i_{n}}}e_{i_{1}}{\left(e_{i_{2}}⊗\cdots ⊗e_{i_{n}}\right)}^{T}\left(e_{i_{2}}⊗\cdots ⊗e_{i_{n}}\right)e_{i_{1}}^{T}∥}_{2}\\ & = & \max_{i_{1}}\sum_{i_{2},\cdots ,i_{n}}\frac{1_{(i_{1},i_{2},\cdots ,i_{n})\in \Omega }}{W_{i_{1}i_{2}\cdots i_{n}}}\leq \mu_{1}^{2}. \end{array}$$

Similarly,

$$\begin{array}{ccc} & & {∥\sum_{i_{1},\cdots ,i_{n}}\frac{1_{(i_{1},\cdots ,i_{n})\in \Omega }}{W_{i_{1}\cdots i_{n}}}\left(e_{i_{2}}⊗\cdots ⊗e_{i_{n}}\right)e_{i_{1}}^{T}e_{i_{1}}{\left(e_{i_{2}}⊗\cdots ⊗e_{i_{n}}\right)}^{T}∥}_{2}\\ & = & \max_{i_{2},\cdots ,i_{n}}\sum_{i_{1}}\frac{1_{(i_{1},i_{2},\cdots ,i_{n})\in \Omega }}{W_{i_{1}i_{2}\cdots i_{n}}}\leq \mu_{1}^{2}. \end{array}$$

By ([61] Theorem 1.5), for any t>0,

$$\begin{array}{c} P\left\{∥{\left(W^{\left(-1/2\right)}⊡Z_{\Omega }\right)}_{\left(1\right)}∥\geq t\right\}\leq \left(d_{1}+\prod_{j\neq 1}d_{j}\right)\exp\left(-\frac{t^{2}}{2\sigma^{2}\mu_{1}^{2}}\right). \end{array}$$

We conclude that with probability at least $1-\frac{1}{d_{1}+\prod_{j\neq 1}d_{j}}$, we have

$$∥{\left(W^{\left(-1/2\right)}⊡Z_{\Omega }\right)}_{\left(1\right)}∥\leq 2\sigma \mu_{1}\sqrt{\log(d_{1}+\prod_{j\neq 1}d_{j})}.$$

Similarly, we have

$$\begin{array}{ccc} & & {∥{\left(W^{\left(-1/2\right)}⊡Y_{\Omega }-W^{\left(1/2\right)}⊡T\right)}_{\left(k\right)}∥}_{2}\\ & \leq & \sqrt{r_{k}}{∥T∥}_{\infty }{∥{\left(W^{\left(-1/2\right)}⊡1_{\Omega }-W^{\left(1/2\right)}\right)}_{\left(k\right)}∥}_{2}+{∥{\left(W^{\left(-1/2\right)}⊡Z_{\Omega }\right)}_{\left(k\right)}∥}_{2}, \end{array}$$

with

$${∥{\left(W^{\left(-1/2\right)}⊡Z_{\Omega }\right)}_{\left(k\right)}∥}_{2}\leq 2\sigma \mu_{k}\sqrt{\log(d_{k}+\prod_{j\neq k}d_{j})}$$

with probability at least $1-\frac{1}{d_{k}+\prod_{j\neq k}d_{j}}$, for k=2,⋯,n. Plugging all these into (A2), we can obtain the bound in our theorem. □

Next we are going to study the special case when the sampling set Ω∼W.

### Appendix B.2. Case Study: Ω∼W

In this section, we would provide upper and lower bounds for the weighted HOSVD algorithm.

#### Appendix B.2.1. Upper Bound

First, let us understand the bounds $\lambda_{\ell }$ and $\mu_{\ell }$ in the case when Ω∼W for ℓ=1,⋯,n.

**Lemma** **A1.**
*Let $W=w_{1}⊗\cdots ⊗w_{n}\in R^{d_{1}\times \cdots \times d_{n}}$ be a CP rank-1 tensor so that all $\left(i_{1},\cdots ,i_{n}\right)\in \left[d_{1}\right]\times \cdots \times \left[d_{n}\right]$ with $W_{i_{1}\cdots i_{n}}\in \left[\frac{1}{\sqrt{\prod_{j=1}^{n}d_{j}}},1\right]$. Suppose that $\Omega \subseteq \left[d_{1}\right]\times \cdots \times \left[d_{n}\right]$ so that for each $i_{1}\in \left[d_{1}\right],\cdots ,i_{n}\in \left[d_{n}\right]$, $(i_{1},\cdots ,i_{n})\in \Omega$ with probability $W_{i_{1}\cdots i_{n}}$, independently for each $\left(i_{1},\cdots ,i_{n}\right)$. Then with probability at least $1-\sum_{\ell =1}^{n}\frac{2}{d_{\ell }+\prod_{j\neq \ell }d_{j}}$ over the choice of Ω, we have for ℓ=1,⋯,n*

(A3) $$\lambda_{\ell }={∥{\left(W^{\left(1/2\right)}-W^{\left(-1/2\right)}⊡1_{\Omega }\right)}_{\left(\ell \right)}∥}_{2}\leq 2\sqrt{d_{\ell }+\prod_{k\neq \ell }d_{k}}\log\left(d_{\ell }+\prod_{k\neq \ell }d_{k}\right),$$

*and*

(A4) $$\mu_{\ell }\leq 2\sqrt{\left(d_{\ell }+\prod_{k\neq \ell }d_{k}\right)\log\left(d_{\ell }+\prod_{k\neq \ell }d_{k}\right)}.$$

**Proof.** Fix $i_{1}\in \left[d_{1}\right]$. Bernstein’s inequality yields

$$\begin{array}{ccc} & & P\left\{\sum_{i_{2},\cdots ,i_{n}}\frac{1_{(i_{1},\cdots ,i_{n})\in \Omega }}{w_{1}\left(i_{1}\right)\cdots w_{n}\left(i_{n}\right)}-\prod_{k\neq 1}d_{k}\geq t\right\}\\ & \leq & \exp\left(\frac{-t^{2}/2}{\sum_{i_{2},\cdots ,i_{n}}\left(\frac{1}{w_{1}\left(i_{1}\right)\cdots w_{n}\left(i_{n}\right)}-1\right)+\frac{1}{3}\sqrt{\prod_{k=1}^{n}d_{k}}t}\right). \end{array}$$

and

$$\begin{array}{ccc} & & P\left\{\sum_{i_{1}}\frac{1_{(i_{1},\cdots ,i_{n})\in \Omega }}{w_{1}\left(i_{1}\right)\cdots w_{n}\left(i_{n}\right)}-d_{1}\geq t\right\}\\ & \leq & \exp\left(\frac{-t^{2}/2}{\sum_{i_{1}}\left(1/(w_{1}\left(i_{1}\right)\cdots w_{n}\left(i_{n}\right))-1\right)+\frac{1}{3}\sqrt{\prod_{k=1}^{n}d_{k}}t}\right). \end{array}$$

Set $t=2\sqrt{2}\left(d_{1}+\prod_{j\neq 1}d_{j}\right)\log\left(d_{1}+\prod_{j\neq 1}d_{j}\right)$, then we have

$$\begin{array}{ccc} & & P\left\{\sum_{i_{2},\cdots ,i_{n}}\frac{1_{(i_{1},i_{2},\cdots ,i_{n})\in \Omega }}{w_{1}\left(i_{1}\right)\cdots w_{n}\left(i_{n}\right)}-\prod_{k\neq 1}d_{k}\geq 2\sqrt{2}\left(d_{1}+\prod_{j\neq 1}d_{j}\right)\log\left(d_{1}+\prod_{j\neq 1}d_{j}\right)\right\}\\ & \leq & 1/{\left(d_{1}+\prod_{j\neq 1}d_{j}\right)}^{2} \end{array}$$

and

$$\begin{array}{ccc} & & P\left\{\sum_{i_{1}}\frac{1_{(i_{1},i_{2},\cdots ,i_{n})\in \Omega }}{w_{1}\left(i_{1}\right)w_{2}\left(i_{2}\right)\cdots w_{n}\left(i_{n}\right)}-d_{1}\geq 2\sqrt{2}\left(d_{1}+\prod_{j\neq 1}d_{j}\right)\log\left(d_{1}+\prod_{j\neq 1}d_{j}\right)\right\}\\ & \leq & 1/{\left(d_{1}+\prod_{j\neq 1}d_{j}\right)}^{2}. \end{array}$$

Hence, by taking a union bound,

$$\begin{array}{ccc} & & P\left\{\max\left\{\max_{i_{1}}\sum_{i_{2},\cdots ,i_{n}}\frac{1_{(i_{1},i_{2},\cdots ,i_{n})\in \Omega }}{w_{1}\left(i_{1}\right)w_{2}\left(i_{2}\right)\cdots w_{n}\left(i_{n}\right)},\max_{i_{2},\cdots ,i_{n}}\sum_{i_{1}}\frac{1_{(i_{1},i_{2},\cdots ,i_{n})\in \Omega }}{w_{1}\left(i_{1}\right)w_{2}\left(i_{2}\right)\cdots w_{n}\left(i_{n}\right)}\right\}\right.\\ & & \left.\geq 4\left(d_{1}+\prod_{j\neq 1}d_{j}\right)\log\left(d_{1}+\prod_{j\neq 1}d_{j}\right)\right\}\leq \frac{1}{d_{1}+\prod_{j\neq 1}d_{j}}. \end{array}$$

Similarly, we have

$$\begin{array}{ccc} P\left\{\mu_{k}^{2}\geq 4\left(d_{k}+\prod_{j\neq k}d_{j}\right)\log\left(d_{k}+\prod_{j\neq k}d_{j}\right)\right\} & \leq & \frac{1}{d_{k}+\prod_{j\neq k}d_{j}},\;\mathrm{for}\;\mathrm{all}\;k=2,\cdots ,n. \end{array}$$

Combining all these inequalities above, with probability at least $1-\sum_{\ell =1}^{n}\frac{1}{d_{\ell }+\prod_{j\neq \ell }d_{j}}$, we have

$$\mu_{\ell }\leq 2\sqrt{\left(d_{\ell }+\prod_{k\neq \ell }d_{k}\right)\log\left(d_{\ell }+\prod_{k\neq \ell }d_{k}\right)},\;\mathrm{for}\;\mathrm{all}\;\ell =1,\cdots ,n.$$

Next we would bound $\lambda_{\ell }$ in (A3). First of all, let’s consider $∥{\left(W^{\left(1/2\right)}-W^{\left(-1/2\right)}⊡1_{\Omega }\right)}_{\left(1\right)}{∥}_{2}$. Set $\gamma_{i_{1}\cdots i_{n}}=\frac{W_{i_{1}\cdots i_{n}}-1_{(i_{1},\cdots ,i_{n})\in \Omega }}{\sqrt{W_{i_{1}\cdots i_{n}}}}$. Then

$${\left(W^{\left(1/2\right)}-W^{\left(-1/2\right)}⊡1_{\Omega }\right)}_{\left(1\right)}=\sum_{i_{1},\cdots ,i_{n}}\gamma_{i_{1}\cdots i_{n}}e_{i_{1}}{\left(e_{i_{2}}⊗\cdots ⊗e_{i_{n}}\right)}^{T}.$$

Notice that

$$\begin{array}{ccc} & & \sum_{i_{1},\cdots ,i_{n}}E\left(\gamma_{i_{1}\cdots i_{n}}^{2}e_{i_{1}}{\left(e_{i_{2}}⊗\cdots ⊗e_{i_{n}}\right)}^{T}\left(e_{i_{2}}⊗\cdots ⊗e_{i_{n}}\right)e_{i_{1}}^{T}\right)\\ & = & \sum_{i_{1}}\left(\sum_{i_{2},\cdots ,i_{n}}E\left(\gamma_{i_{1}\cdots i_{n}}^{2}\right)\right)e_{i_{1}}e_{i_{1}}^{T}. \end{array}$$

Since $E\left(\gamma_{i_{1}\cdots i_{n}}^{2}\right)=1-W_{i_{1}\cdots i_{n}}\leq 1-\frac{1}{\sqrt{d_{1}\cdots d_{n}}}\leq 1$, then

$${∥\sum_{i_{1},\cdots ,i_{n}}E\left(\gamma_{i_{1}\cdots i_{n}}^{2}e_{i_{1}}{\left(e_{i_{2}}⊗\cdots ⊗e_{i_{n}}\right)}^{T}\left(e_{i_{2}}⊗\cdots ⊗e_{i_{n}}\right)e_{i_{1}}^{T}\right)∥}_{2}\leq \prod_{j\neq 1}d_{j}.$$

Similarly,

$${∥\sum_{i_{1},\cdots ,i_{n}}E\left(\gamma_{i_{1}\cdots i_{n}}^{2}\left(e_{i_{2}}⊗\cdots ⊗e_{i_{n}}\right)e_{i_{1}}^{T}e_{i_{1}}{\left(e_{i_{2}}⊗\cdots ⊗e_{i_{n}}\right)}^{T}\right)∥}_{2}\leq d_{1}.$$

In addition,

$${∥\gamma_{i_{1}\cdots i_{n}}e_{i_{1}}{\left(e_{i_{2}}⊗\cdots ⊗e_{i_{n}}\right)}^{T}∥}_{2}\leq {\left(\prod_{j=1}^{n}d_{j}\right)}^{1/4}\leq \sqrt{\frac{d_{1}+\prod_{j\neq 1}d_{j}}{2}}.$$

Then, the matrix Bernstein Inequality ([61] Theorem 1.4) gives

$$\begin{array}{cc} \; & \;P\left\{{∥{\left(W^{\left(1/2\right)}-W^{\left(-1/2\right)}⊡1_{\Omega }\right)}_{\left(1\right)}∥}_{2}\geq t\right\}\\ \leq & \;\left(d_{1}+\prod_{j\neq 1}d_{j}\right)\exp\left(-\frac{t^{2}/2}{\left(d_{1}+\prod_{j\neq 1}d_{j}\right)+\frac{t}{3}\sqrt{\left(d_{1}+\prod_{j\neq 1}d_{j}\right)/2}}\right). \end{array}$$

Let $t=2\sqrt{d_{1}+\prod_{j\neq 1}d_{j}}\log\left(d_{1}+\prod_{j\neq 1}d_{j}\right)$, then we have

$$\begin{array}{c} P\left\{{∥{\left(W^{\left(1/2\right)}-W^{\left(-1/2\right)}⊡1_{\Omega }\right)}_{\left(1\right)}∥}_{2}\geq 2\sqrt{d_{1}+\prod_{j\neq 1}d_{j}}\log\left(d_{1}+\prod_{j\neq 1}d_{j}\right)\right\}\leq \frac{1}{d_{1}+\prod_{j\neq 1}d_{j}}. \end{array}$$

Similarly,

$$\begin{array}{c} P\left\{{∥{\left(W^{\left(1/2\right)}-W^{\left(-1/2\right)}⊡1_{\Omega }\right)}_{\left(k\right)}∥}_{2}\geq 2\sqrt{d_{k}+\prod_{j\neq k}d_{k}}\log\left(d_{k}+\prod_{j\neq k}d_{j}\right)\right\}\leq \frac{1}{d_{k}+\prod_{j\neq k}d_{j}}, \end{array}$$

for all k=2,⋯,n. Thus, with probability at least $1-\sum_{\ell =1}^{n}\frac{1}{d_{\ell }+\prod_{j\neq \ell }d_{j}}$, we have

$${∥{\left(W^{\left(1/2\right)}-W^{\left(-1/2\right)}⊡1_{\Omega }\right)}_{\left(\ell \right)}∥}_{2}\leq 2\sqrt{d_{\ell }+\prod_{k\neq \ell }d_{k}}\log\left(d_{\ell }+\prod_{k\neq \ell }d_{k}\right),\;\mathrm{for}\;\mathrm{all}\;\ell =1,\cdots ,n.$$

By a union of bounds in (A4) and (A3), we could establish the lemma. □

**Lemma** **A2.**
*Let $m={∥W^{\left(1/2\right)}∥}_{F}^{2}$. Then with probability at least 1−2exp(−3m/104), over the choice of Ω*

$$\left||\Omega |-m\right|\leq \frac{m}{4}.$$

**Proof.** Please note that

$$\left||\Omega |-m\right|=\left|\sum_{i_{1},\cdots ,i_{n}}\left(1_{(i_{1},\cdots ,i_{n})\in \Omega }-W_{i_{1}\cdots i_{n}}\right)\right|=\left|\sum_{i_{1},\cdots ,i_{n}}(1_{(i_{1},\cdots ,i_{n})\in \Omega }-E\left(1_{(i_{1},\cdots ,i_{n})\in \Omega }\right)\right|,$$

which is the sum of zero-mean independent random variables. Observe that $|1_{(i_{1},\cdots ,i_{n})\in \Omega }-E\left(1_{(i_{1},\cdots ,i_{n})\in \Omega }\right)\left|=\right|1_{(i_{1},\cdots ,i_{n})\in \Omega }-W_{i_{1}\cdots i_{n}}|\leq 1$ and

$$\sum_{i_{1},\cdots ,i_{n}}E{\left(1_{(i_{1},\cdots ,i_{n})\in \Omega }-W_{i_{1}\cdots i_{n}}\right)}^{2}=\sum_{i_{1},\cdots ,i_{n}}\left(W_{i_{1}\cdots i_{n}}-W_{i_{1}\cdots i_{n}}^{2}\right)\leq m.$$

By Bernstein’s inequality,

$$\begin{array}{ccc} P\left(||\Omega |-m|\geq t\right) & \leq & 2\exp\left(-\frac{t^{2}/2}{m+t/3}\right). \end{array}$$

Set t=m/4, then we have

$$\begin{array}{ccc} P\left(||\Omega |-m|\geq m/4\right) & \leq & 2\exp\left(-\frac{m^{2}/32}{m+m/12}\right)=2\exp\left(-3m/104\right). \end{array}$$

□

Next let us give the formal statement for the upper bounds in Theorem 3.
**Theorem** **A2.** *Let $W=w_{1}⊗\cdots ⊗w_{n}\in R^{d_{1}\times \cdots \times d_{n}}$ be a CP rank-1 tensor so that for all $\left(i_{1},\cdots ,i_{n}\right)\in \left[d_{1}\right]\times \cdots \times \left[d_{n}\right]$ we have $W_{i_{1}\cdots i_{n}}\in \left[\frac{1}{\sqrt{d_{1}\cdots d_{n}}},1\right]$. Suppose that we choose each $\left(i_{1},\cdots ,i_{n}\right)\in \left[d_{1}\right]\times \cdots \times \left[d_{n}\right]$ independently with probability $W_{i_{1}\cdots i_{n}}$ to form a set $\Omega \subseteq \left[d_{1}\right]\times \cdots \times \left[d_{n}\right]$. Then with probability at least*

$$1-2\exp\left(-\frac{3}{104}\sqrt{\prod_{j=1}^{n}d_{j}}\;\right)-\sum_{k=1}^{n}\frac{2}{d_{k}+\prod_{j\neq k}d_{j}}$$

*For the weighted HOSVD Algorithm named A, A returns $\hat{T}=A\left(T_{\Omega }+Z_{\Omega }\right)$ for any Tucker rank r tensor T with ${∥T∥}_{\infty }\leq \beta$ so that with probability at least $1-\sum_{k=1}^{n}\frac{1}{d_{k}+\prod_{j\neq k}d_{j}}$ over the choice of Z,*

$$\begin{array}{ccc} \frac{{∥W^{\left(1/2\right)}⊡\left(T-\hat{T}\right)∥}_{F}}{{∥W^{\left(1/2\right)}∥}_{F}} & \leq & \frac{\sqrt{5}\beta }{\sqrt{\left|\Omega \right|}}\left(\sum_{k=1}^{n}3r_{k}\sqrt{d_{k}+\prod_{j\neq k}d_{j}}\log\left(d_{k}+\prod_{j\neq k}d_{j}\right)\right)\\ & & +\frac{\sqrt{5}\sigma }{\left|\Omega \right|}\left(\sum_{k=1}^{n}6\sqrt{r_{k}\left(d_{k}+\prod_{j\neq k}d_{j}\right)}\log\left(d_{k}+\prod_{j\neq k}d_{j}\right)\right) \end{array}$$

**Proof.** This is directly from Theorem A1, Lemmas A1 and A2. □

#### Appendix B.2.2. Lower Bound

To deduce the lower bound, we have to construct a finite subset *S* in the cone $K_{r}$ so that we can approximate the minimal distance between two different elements in *S*. Before we prove the lower bound, we need the following theorems and lemmas.
**Theorem** **A3** (Hanson-Wright inequality). *There is some constant c>0 so that the following holds. Let $\xi \in {\left\{0,\pm 1\right\}}^{d}$ be a vector with mean-zero, independent entries, and let F be any matrix which has zero diagonal. Then*

$$P\left\{|\xi^{T}F\xi |>t\right\}\leq 2\exp\left(-c\cdot \min\left\{\frac{t^{2}}{{∥F∥}_{F}^{2}},\frac{t}{{∥F∥}_{2}}\right\}\right).$$

**Theorem** **A4** (Fano’s Inequality). *Let $F=\{f_{0},\cdots ,f_{n}\}$ be a collection of densities on K, and suppose that A:K→{0,⋯,n}. Suppose there is some β>0 such that for any i≠j, $D_{KL}\left(f_{i}∥f_{j}\right)\leq \beta$. Then*

$$\max_{i}P_{K∼f_{i}}\left\{A(K)\neq i\right\}\geq 1-\frac{\beta +\log(2)}{\log(n)}.$$

The following lemma specializes Fano’s Inequality to our setting, which is a generalization of ([19] Lemma 19). In the following lemma, we show that for any reconstruction algorithm on a set $K\subseteq R^{d_{1}\times \cdots \times d_{n}}$, with probability no less than $\frac{1}{2}$, there exists some elements in *K* such that the weighted reconstruction error is bounded below by some quantity, where the quantity is independent of the algorithm.
**Lemma** **A3.** *Let $K\subseteq R^{d_{1}\times \cdots \times d_{n}}$, and let S⊆K be a finite subset of K so that |S|>16. Let $\Omega \subseteq \left[d_{1}\right]\times \cdots \times \left[d_{n}\right]$ be a sampling pattern. Let σ>0 and choose*

$$\kappa \leq \frac{\sigma \sqrt{\log|S|}}{4\max_{T\in S}{∥T_{\Omega }∥}_{F}},$$

*and suppose that*

κS⊆K.

*Let $Z\in R^{d_{1}\times \cdots \times d_{n}}$ be a tensor whose entries $Z_{i_{1}\cdots i_{n}}$ are i.i.d., $Z_{i_{1}\cdots i_{n}}∼N\left(0,\sigma^{2}\right)$. Let $H\subseteq R^{d_{1}\times \cdots \times d_{n}}$ be any weight tensor.* *Then for any algorithm $A:R^{\Omega }\to R^{d_{1}\times \cdots \times d_{n}}$ that takes as input $T_{\Omega }+Z_{\Omega }$ for T∈K and outputs an estimate $\hat{T}$ to T, there is some X∈K so that*

(A5) $${∥H⊡(A\left(X_{\Omega }+Z_{\Omega }\right)-X)∥}_{F}\geq \frac{\kappa }{2}\min_{T\neq T^{\prime }\in S}{∥H⊡(T-T^{\prime })∥}_{F}$$

*with probability at least $\frac{1}{2}$.*
**Proof.** Consider the set

$$S^{\prime }=\kappa S=\left\{\kappa T:T\in S\right\}$$

which is a scaled version of *S*. By our assumption, $S^{\prime }\subseteq K$. Recall that the Kullback–Leibler (KL) divergence between two multivariate Gaussians is given by

$$\begin{array}{ccc} & & D_{KL}\left(N\left(\mu_{1},\Sigma_{1}\right)∥N\left(\mu_{2},\Sigma_{2}\right)\right)\\ & = & \frac{1}{2}\left(\log\left(\frac{\det(\Sigma_{2})}{\det(\Sigma_{1})}\right)-n+tr\left(\Sigma_{2}^{-1}\Sigma_{1}\right)+\langle \Sigma_{2}^{-1}\left(\mu_{2}-\mu_{1}\right),\mu_{2}-\mu_{1}\rangle \right), \end{array}$$

where μ${}_{1}$, μ${}_{2}\in R^{n}$. Specializing to $U,V\in S^{\prime }$, with $I=I_{\Omega \times \Omega }$

$$\begin{array}{ccc} D_{KL}\left(U_{\Omega }+Z_{\Omega }∥V_{\Omega }+Z_{\Omega }\right) & = & D_{KL}\left(N\left(U_{\Omega },\sigma^{2}I\right)∥N\left(V_{\Omega },\sigma^{2}I\right)\right)\\ & = & \frac{{∥U_{\Omega }-V_{\Omega }∥}_{F}^{2}}{2\sigma^{2}}\\ & \leq & \max_{T\in S^{\prime }}\frac{2{∥T_{\Omega }∥}_{F}^{2}}{\sigma^{2}}=\frac{2\kappa^{2}}{\sigma^{2}}\max_{T\in S}{∥T_{\Omega }∥}_{F}^{2}. \end{array}$$

Suppose that A is as in the statement of the lemma. Define an algorithm $\bar{A}:R^{\Omega }\to R^{d_{1}\times \cdots \times d_{n}}$ so that for any $Y\in R^{\Omega }$ if there exists $T\in S^{\prime }$ such that

$${∥H⊡(T-A(Y))∥}_{F}<\frac{1}{2}\min_{T\neq T^{\prime }\in S^{\prime }}{∥H⊡\left(T-T^{\prime }\right)∥}_{F}:=\frac{\rho }{2},$$

then set $\bar{A}\left(Y\right)=T$ (notice that if such T exists, then it is unique), otherwise, set $\bar{A}\left(Y\right)=A\left(Y\right)$. Then by the Fano’s inequality, there is some $T\in S^{\prime }$ so that

$$\begin{array}{ccc} P\left\{\bar{A}\left(T_{\Omega }+Z_{\Omega }\right)\neq T\right\} & \geq & 1-\frac{2\max_{T\in S^{\prime }}{∥T_{\Omega }∥}_{F}^{2}}{\sigma^{2}\log\left(|S|-1\right)}-\frac{\log(2)}{\log(|S|-1)}\\ & = & 1-\frac{2\kappa^{2}\max_{T\in S}{∥T_{\Omega }∥}_{F}^{2}}{\sigma^{2}\log\left(|S|-1\right)}-\frac{\log(2)}{\log(|S|-1)}\\ & \geq & 1-\frac{1}{4}-\frac{1}{4}=\frac{1}{2}. \end{array}$$

If $\bar{A}\left(T_{\Omega }+Z_{\Omega }\right)\neq T$, then $∥H⊡\left(A\left(T_{\Omega }+Z_{\Omega }\right)-T\right){∥}_{F}>\rho /2$, and so

$$P\left\{∥H⊡\left(A\left(T_{\Omega }+Z_{\Omega }\right)-T\right){∥}_{F}\geq \rho /2\right\}\geq P\left\{\bar{A}\left(T_{\Omega }+Z_{\Omega }\right)\neq T\right\}\geq 1/2.$$

Finally, we observe that

$$\frac{\rho }{2}=\frac{1}{2}\min_{T\neq T^{\prime }\in S^{\prime }}∥H⊡\left(T-T^{\prime }\right){∥}_{F}=\frac{\kappa }{2}\min_{T\neq T^{\prime }\in S}{∥H⊡\left(T-T^{\prime }\right)∥}_{F},$$

which completes the proof. □

To understand the lower bound $\frac{\kappa }{2}\min_{T\neq T\in S}{∥H⊡\left(T-T^{\prime }\right)∥}_{F}$ in (A5), we construct a specific finite subset *S* for the cone of Tucker rank r tensors in the following lemma.
**Lemma** **A4.** *There is some constant c so that the following holds. Let $d_{1},\cdots ,d_{n}>0$ and $r_{1},\cdots ,r_{n}>0$ be sufficiently large. Let K be the cone of Tucker rank r tensors with $r=[r_{1}\;\cdots \;r_{n}]$, H be any CP rank-1 weight tensor, and B be any CP rank-1 tensor with ${∥B∥}_{\infty }\leq 1$. Write $H=h_{1}⊗\cdots ⊗h_{n}$ and $B=b_{1}⊗\cdots ⊗b_{n}$, and*

$$w_{1}={\left(h_{1}⊡b_{1}\right)}^{\left(2\right)},\cdots ,w_{n}={\left(h_{n}⊡b_{n}\right)}^{\left(2\right)}.$$

*Let*

$$\gamma =\sqrt{\frac{1}{2}\left(\prod_{k=1}^{n}r_{k}\right)\log\left(8\prod_{k=1}^{n}d_{k}\right)}.$$

*There is a set $S\subseteq K\cap \gamma B_{\infty }$ so that*

1. *The set has size |S|≥N, for*
  $$\begin{array}{c} N=C\exp\left(c\cdot \min\left\{\frac{\prod_{k=1}^{n}r_{k}}{\left(\prod_{k=1}^{n}(2r_{k}(∥w_{k}{∥}_{2}/∥w_{k}{∥}_{1}{)}^{2}+1)\right)-1},\prod_{k=1}^{n}r_{k},\right.\right.\\ \left.\left.\frac{\prod_{k=1}^{n}r_{k}}{\left(\prod_{k=1}^{n}(2∥w_{k}{∥}_{2}/∥w_{k}{∥}_{1}\sqrt{r_{k}\log\left(r_{k}\right)}+2∥w_{k}{∥}_{\infty }/∥w_{k}{∥}_{1}r_{k}\log\left(r_{k}\right)+1)\right)-1}\right\}\right). \end{array}$$
2. *$∥T_{\Omega }{∥}_{F}\leq 2\sqrt{\prod_{k=1}^{n}r_{k}}{∥B_{\Omega }∥}_{F}$ for all T∈S.*
3. *${∥H⊡(T-\tilde{T})∥}_{F}\geq \sqrt{\prod_{k=1}^{n}r_{k}}{∥H⊡B∥}_{F}$ for all $T\neq \tilde{T}\in S$.*

**Proof.** Let $\Psi \subseteq {\left\{\pm 1\right\}}^{r_{1}\times \cdots \times r_{n}}$ be a set of random ±1-valued tensors chosen uniformly at random with replacement, of size 4N. Choose ${}^{i}U\in {\left\{\pm 1\right\}}^{d_{i}\times r_{i}}$ to be determined below for all i=1,⋯,n. Let

$$S=\left\{B⊡(C\times_{1}{}^{1}U\times_{2}\cdots \times_{n}{}^{n}U):C\in \Psi \right\}.$$

First of all, we would estimate ${∥T_{\Omega }∥}_{F}$ and ${∥T∥}_{\infty }$. Please note that

$$\begin{array}{ccc} E{∥T_{\Omega }∥}_{F}^{2} & = & E\sum_{(i_{1},\cdots ,i_{n})\in \Omega }B_{i_{1}\cdots i_{n}}^{2}{\left(\sum_{j_{1},\cdots ,j_{n}}C_{j_{1}\cdots j_{n}}{}^{1}U\left(i_{1},j_{1}\right)\cdots {}^{n}U\left(i_{n},j_{n}\right)\right)}^{2}=\left(\prod_{i=1}^{n}r_{i}\right){∥B_{\Omega }∥}_{F}^{2}, \end{array}$$

where the expectation is over the random choice of C. Then by Markov’s inequality,

$$P\left\{∥T_{\Omega }{∥}_{F}^{2}\geq \left(4\prod_{i=1}^{n}r_{i}\right){∥B_{\Omega }∥}_{F}^{2}\right\}\leq \frac{1}{4}.$$

We also have

$${∥T∥}_{\infty }=\max_{i_{1},\cdots ,i_{n}}\left|B_{i_{1}\cdots i_{n}}\right|\left|\sum_{j_{1},\cdots ,j_{n}}C_{j_{1}\cdots j_{n}}{}^{1}U\left(i_{1},j_{1}\right)\cdots {}^{n}U\left(i_{n},j_{n}\right)\right|.$$

By Hoeffding’s inequality, we have

$$\begin{array}{ccc} P\left\{\left|\sum_{j_{1},\cdots ,j_{n}}C_{j_{1}\cdots j_{n}}{}^{1}U\left(i_{1},j_{1}\right)\cdots {}^{n}U\left(i_{n},j_{n}\right)\right|\geq t\right\} & \leq & 2\exp\left(-\frac{2t^{2}}{\prod_{k=1}^{n}r_{k}}\right). \end{array}$$

Using the fact that $|B_{i_{1}\cdots i_{n}}|\leq 1$ and a union bound over all $\prod_{k=1}^{n}d_{k}$ values of $i_{1},\cdots ,i_{n}$, we conclude that

$$\begin{array}{ccc} & & P\left\{{∥T∥}_{\infty }\geq \sqrt{\frac{1}{2}\left(\prod_{k=1}^{n}r_{k}\right)\log\left(8\prod_{k=1}^{n}d_{k}\right)}\right\}\\ & \leq & \left(\prod_{k=1}^{n}d_{k}\right)P\left\{\left|\sum_{j_{1},\cdots ,j_{n}}C_{j_{1}\cdots j_{n}}{}^{1}U\left(i_{1},j_{1}\right)\cdots {}^{n}U\left(i_{n},j_{n}\right)\right|\geq \sqrt{\frac{1}{2}\left(\prod_{k=1}^{n}r_{k}\right)\log\left(8\prod_{k=1}^{n}d_{k}\right)}\right\}\\ & \leq & \frac{1}{4}. \end{array}$$

Thus, for a tensor T∈S, the probability that both of ${∥T∥}_{\infty }\leq \sqrt{\frac{1}{2}\left(\prod_{k=1}^{n}r_{k}\right)\log\left(8\prod_{k=1}^{n}d_{k}\right)}$ and $∥T_{\Omega }{∥}_{F}\leq 2\sqrt{\prod_{k=1}^{n}r_{k}}{∥B_{\Omega }∥}_{F}$ hold is at least $\frac{1}{2}$. Thus, by a Chernoff bound it follows that with probability at least 1−exp(−CN) for some constant *C*, there are at least $\frac{\left|S\right|}{4}$ tensors T∈S such that all of these hold. Let $\tilde{S}\subseteq S$ be the set of such T’s. The set guaranteed in the statement of the lemma will be $\tilde{S}$, which satisfies both item 1 and 2 in the lemma and is also contained in $K\cap \gamma B_{\infty }$. Thus, we consider item 3: we are going to show that this holds for *S* with high probability, thus in particularly it will hold for $\tilde{S}$, and this will complete the proof of the lemma. Fix $T\neq \tilde{T}\in S$, and write

$$\begin{array}{ccc} & & {∥H⊡(T-\tilde{T})∥}_{F}^{2}\\ & = & {∥H⊡B⊡(\left(C-\tilde{C}\right)\times_{1}{}^{1}U\times_{2}\cdots \times_{n}{}^{n}U)∥}_{F}^{2}\\ & = & \sum_{i_{1},\cdots ,i_{n}}H_{i_{1}\cdots i_{n}}^{2}B_{i_{1}\cdots i_{n}}^{2}{\left(\sum_{j_{1},\cdots ,j_{n}}\left(C_{j_{1}\cdots j_{n}}-{\tilde{C}}_{j_{1}\cdots j_{n}}\right){}^{1}U\left(i_{1},j_{1}\right)\cdots {}^{n}U\left(i_{n},j_{n}\right)\right)}^{2}\\ & = & 4\sum_{i_{1},\cdots ,i_{n}}H_{i_{1}\cdots i_{n}}^{2}B_{i_{1}\cdots i_{n}}^{2}{\langle \xi ,{}^{1}U\left(i_{1},:\right)⊗\cdots ⊗{}^{n}U\left(i_{n},:\right)\rangle }^{2}, \end{array}$$

where ξ is the vectorization of $\frac{1}{2}\left(C-\tilde{C}\right)$. Thus, each entry of ξ is independently 0 with probability $\frac{1}{2}$ or ±1 with probability $\frac{1}{4}$ each. Rearranging the terms, we have

(A6) $$\begin{array}{ccc} {∥H⊡(T-\tilde{T})∥}_{F}^{2} & = & 4\xi^{T}{\left({}^{1}U⊗\cdots ⊗{}^{n}U\right)}^{T}\left(D_{1}⊗\cdots ⊗D_{n}\right)\left({}^{1}U⊗\cdots ⊗{}^{n}U\right)\xi \\ & = & 4\xi^{T}\left(\left({}^{1}U^{T}D_{1}{}^{1}U\right)⊗\cdots ⊗\left({}^{n}U^{T}D_{n}{}^{n}U\right)\right)\xi \\ & = & 4\xi^{T}\left({⊗}_{k=1}^{n}\left({}^{k}U^{T}D_{k}{}^{k}U\right)\right)\xi , \end{array}$$

where $D_{k}$ denotes the $d_{k}\times d_{k}$ diagonal matrix with $w_{k}$ on the diagonal. To understand (A6), we need to understand the matrix ${⊗}_{k=1}^{n}\left({}^{k}U^{T}D_{k}{}^{k}U\right)\in R^{\prod_{k=1}^{n}r_{k}\times \prod_{k=1}^{n}r_{k}}$. The diagonal of this matrix is $\left(\prod_{k=1}^{n}{∥w_{k}∥}_{1}\right)I$. We will choose the matrix ${}^{k}U$ for k=1,⋯,n so that the off-diagonal terms are small. □
**Theorem** **A5.** *There are matrices ${}^{k}U\in {\left\{\pm 1\right\}}^{d_{k}\times r_{k}}$ for k=1,⋯,n such that:*

(a) $$\begin{array}{c} {∥\left({⊗}_{k=1}^{n}\left({}^{k}U^{T}D_{k}{}^{k}U\right)\right)-\left(\prod_{j=1}^{n}{∥w_{j}∥}_{1}\right)I∥}_{F}^{2}\leq \left(\prod_{k=1}^{n}\left(2r_{k}^{2}∥w_{k}{∥}_{2}^{2}+r_{k}{∥w_{k}∥}_{1}^{2}\right)\right)-\prod_{k=1}^{n}\left(r_{k}{∥w_{k}∥}_{1}^{2}\right). \end{array}$$
(b) $$\begin{array}{ccc} & & {∥\left({⊗}_{k=1}^{n}\left({}^{k}U^{T}D_{k}{}^{k}U\right)\right)-\left(\prod_{j=1}^{n}{∥w_{j}∥}_{1}\right)I∥}_{2}\\ & \leq & \max\left\{\prod_{k=1}^{n}\left(2{∥w_{k}∥}_{2}\sqrt{r_{k}\log\left(r_{k}\right)}+2{∥w_{k}∥}_{\infty }r_{k}\log\left(r_{k}\right)+{∥w_{k}∥}_{1}\right)-\prod_{k=1}^{n}{∥w_{k}∥}_{1},\prod_{k=1}^{n}{∥w_{k}∥}_{1}\right\}. \end{array}$$

**Proof.** By ([19] Claim 22), there exist matrices ${}^{k}U\in {\left\{\pm 1\right\}}^{d_{k}\times r_{k}}$ such that:

(a) ${∥{}^{k}U^{T}D_{k}{}^{k}U∥}_{F}^{2}\leq 2r_{k}^{2}{∥w_{k}∥}_{2}^{2}+r_{k}{∥w_{k}∥}_{1}^{2}$ and
(b) ${∥{}^{k}U^{T}D_{k}{}^{k}U∥}_{2}\leq 2{∥w_{k}∥}_{2}\sqrt{r_{k}\log\left(r_{k}\right)}+2{∥w_{k}∥}_{\infty }r_{k}\log\left(r_{k}\right)+{∥w_{k}∥}_{1}$.

Using (a) and the fact that ${∥{⊗}_{k=1}^{n}\left({}^{k}U^{T}D_{k}{}^{k}U\right)∥}_{F}^{2}=\prod_{k=1}^{n}{∥{}^{k}U^{T}D_{k}{}^{k}U∥}_{F}^{2}$, we have

$$\begin{array}{ccc} & & {∥\left({⊗}_{k=1}^{n}\left({}^{k}U^{T}D_{k}{}^{k}U\right)\right)-\left(\prod_{k=1}^{n}{∥w_{j}∥}_{1}\right)I∥}_{F}^{2}\\ & = & {∥{⊗}_{k=1}^{n}\left({}^{k}U^{T}D_{k}{}^{k}U\right)∥}_{F}^{2}-{∥\left(\prod_{k=1}^{n}{∥w_{j}∥}_{1}\right)I∥}_{F}^{2}\\ & \leq & \left(\prod_{k=1}^{n}\left(2r_{k}^{2}∥w_{k}{∥}_{2}^{2}+r_{k}{∥w_{k}∥}_{1}^{2}\right)\right)-\prod_{k=1}^{n}\left(r_{k}{∥w_{k}∥}_{1}^{2}\right). \end{array}$$

By (b) and the fact that ${∥{⊗}_{k=1}^{n}\left({}^{k}U^{T}D_{k}{}^{k}U\right)∥}_{2}=\prod_{k=1}^{n}{∥{}^{k}U^{T}D_{k}{}^{k}U∥}_{2}$ (see [62]), we have

$$\begin{array}{cc} \; & \;{∥\left({⊗}_{k=1}^{n}\left({}^{k}U^{T}D_{k}{}^{k}U\right)\right)-\left(\prod_{k=1}^{n}{∥w_{k}∥}_{1}\right)I∥}_{2}\\ \leq \; & \max\left\{\prod_{k=1}^{n}{∥w_{k}∥}_{1},\right.\left.\left(\prod_{k=1}^{n}\left(2∥w_{k}{∥}_{2}\sqrt{r_{k}\log\left(r_{k}\right)}+2∥w_{k}{∥}_{\infty }r_{k}\log\left(r_{k}\right)+{∥w_{k}∥}_{1}\right)\right)-\prod_{k=1}^{n}{∥w_{k}∥}_{1}\right\}. \end{array}$$

□

Having chosen matrices ${}^{k}U$ for k=1,⋯,n, we can now analyze the expression (A6).
**Theorem** **A6.** *There are constants $c,c^{\prime }$ so that with probability at least*

$$\begin{array}{ccc} & & 1-2\exp\left(-c^{\prime \prime }\prod_{k=1}^{n}r_{k}\right)-2\exp\left(-c^{\prime }\cdot \min\left\{\frac{\prod_{k=1}^{n}(r_{k}∥w_{k}{∥}_{1}^{2})}{\prod_{k=1}^{n}(2r_{k}∥w_{k}{∥}_{2}^{2}+∥w_{k}{∥}_{1}^{2})-\prod_{k=1}^{n}{∥w_{k}∥}_{1}^{2}},\right.\right.\\ & & \left.\left.\prod_{k=1}^{n}r_{k},\frac{\prod_{k=1}^{n}(r_{k}∥w_{k}{∥}_{1})}{\left(\prod_{k=1}^{n}(2∥w_{k}{∥}_{2}\sqrt{r_{k}\log\left(r_{k}\right)}+2∥w_{k}{∥}_{\infty }r_{k}\log\left(r_{k}\right)+∥w_{k}{∥}_{1})\right)-\prod_{k=1}^{n}{∥w_{k}∥}_{1}}\right\}\right), \end{array}$$

*we have*

$${∥H⊡\left(T-\tilde{T}\right)\prod_{k=1}^{n}{∥w_{k}∥}_{1}∥}_{F}^{2}\geq \prod_{k=1}^{n}\left(r_{k}{∥w_{k}∥}_{1}\right).$$

**Proof.** We break ${∥H⊡(T-\tilde{T})∥}_{F}^{2}$ into two terms:

$$\begin{array}{ccc} & & {∥H⊡(T-\tilde{T})∥}_{F}^{2}\\ & = & 4\xi^{T}\left({⊗}_{k=1}^{n}{}^{k}U^{T}D_{k}{}^{k}U\right)\xi \\ & = & 4\xi^{T}\left({⊗}_{k=1}^{n}\left({}^{k}U^{T}D_{k}{}^{k}U\right)-\left(\prod_{k=1}^{n}{∥w_{k}∥}_{1}\right)I\right)\xi +4\left(\prod_{k=1}^{n}{∥w_{k}∥}_{1}\right)\xi^{T}\xi \\ & := & (I)+(II). \end{array}$$

For the first term (I), we will use the Hanson-Wright Inequality (see Theorem A3). In our case, the matrix $F=4\left({⊗}_{k=1}^{n}\left({}^{k}U^{T}D_{k}{}^{k}U\right)-\left(\prod_{k=1}^{n}{∥w_{k}∥}_{1}\right)I\right)$. The Frobenius norm of this matrix is bounded by

$$\begin{array}{ccc} {∥F∥}_{F}^{2} & \leq & 16\left(\prod_{k=1}^{n}\left(2r_{k}^{2}∥w_{k}{∥}_{2}^{2}+r_{k}{∥w_{k}∥}_{1}^{2}\right)-\prod_{k=1}^{n}\left(r_{k}{∥w_{k}∥}_{1}^{2}\right)\right). \end{array}$$

The operator norm of *F* is bounded by

$$\begin{array}{ccc} & & {∥F∥}_{2}\\ & \leq & 4\max\left\{\prod_{k=1}^{n}(2∥w_{k}{∥}_{2}\sqrt{r_{k}\log\left(r_{k}\right)}+2∥w_{k}{∥}_{\infty }r_{k}\log\left(r_{k}\right)+∥w_{k}{∥}_{1})-\prod_{k=1}^{n}∥w_{k}{∥}_{1},\prod_{k=1}^{n}{∥w_{k}∥}_{1}\right\}. \end{array}$$

Thus, the Hanson-Wright inequality implies that

$$\begin{array}{ccc} & & P\left\{(I)\geq t\right\}\\ \; & \leq & 2\exp\left(-c\cdot \min\left\{\frac{t^{2}}{16\prod_{k=1}^{n}\left(2r_{k}^{2}∥w_{k}{∥}_{2}^{2}+r_{k}{∥w_{k}∥}_{1}^{2}\right)-16\prod_{k=1}^{n}\left(r_{k}{∥w_{k}∥}_{1}^{2}\right)},\frac{t}{4\prod_{k=1}^{n}{∥w_{k}∥}_{1}},\right.\right.\\ & & \left.\left.\frac{t}{4\left(\prod_{k=1}^{n}(2∥w_{k}{∥}_{2}\sqrt{r_{k}\log\left(r_{k}\right)}+2∥w_{k}{∥}_{\infty }r_{k}\log\left(r_{k}\right)+∥w_{k}{∥}_{1})-\prod_{k=1}^{n}{∥w_{k}∥}_{1}\right)}\right\}\right). \end{array}$$

Plugging in $t=\frac{1}{2}\prod_{k=1}^{n}r_{k}{∥w_{k}∥}_{1}$, and replacing the constant *c* with a different constant $c^{\prime }$, we have

(A7) $$\begin{array}{ccc} & & P\left\{\left(I\right)\geq \frac{1}{2}\prod_{k=1}^{n}r_{k}{∥w_{k}∥}_{1}\right\}\\ & \leq & 2\exp\left(-c^{\prime }\cdot \min\left\{\frac{\prod_{k=1}^{n}r_{k}}{\left(\prod_{k=1}^{n}(2r_{k}(∥w_{k}{∥}_{2}/∥w_{k}{∥}_{1}{)}^{2}+1)\right)-1},\prod_{k=1}^{n}r_{k},\right.\right.\\ & & \left.\left.\frac{\prod_{k=1}^{n}r_{k}}{\left(\prod_{k=1}^{n}(2∥w_{k}{∥}_{2}/∥w_{k}{∥}_{1}\sqrt{r_{k}\log\left(r_{k}\right)}+2∥w_{k}{∥}_{\infty }/∥w_{k}{∥}_{1}r_{k}\log\left(r_{k}\right)+1)\right)-1}\right\}\right). \end{array}$$

Next we turn to the second term (II). We write

$$\left(II\right)=4\left(\prod_{k=1}^{n}{∥w_{k}∥}_{1}\right)\xi^{T}\xi =2\prod_{k=1}^{n}(r_{k}∥w_{k}{∥}_{1})+4\left(\prod_{k=1}^{n}{∥w_{k}∥}_{1}\right)\left({∥\xi ∥}_{2}^{2}-\frac{1}{2}\prod_{k=1}^{n}r_{k}\right)$$

and bound the error term $4\left(\prod_{k=1}^{n}{∥w_{k}∥}_{1}\right)\left({∥\xi ∥}_{2}^{2}-\frac{1}{2}\prod_{k=1}^{n}r_{k}\right)$ with high probability. Observe that ${∥\xi ∥}_{2}^{2}-\frac{1}{2}\prod_{k=1}^{n}r_{k}$ is a zero-mean subgaussian random variable, and thus satisfies for all *t* > 0 that

$$P\left\{\left|{∥\xi ∥}_{2}^{2}-\frac{1}{2}\prod_{k=1}^{n}r_{k}\right|\geq t\right\}\leq 2\exp\left(\frac{-c^{\prime \prime }t^{2}}{\prod_{k=1}^{n}r_{k}}\right)$$

for some constant $c^{\prime \prime }$. Thus, for any t>0 we have

$$P\left\{\left|4\left(\prod_{k=1}^{n}{∥w_{k}∥}_{1}\right)\left({∥\xi ∥}_{2}^{2}-\frac{1}{2}\prod_{k=1}^{n}r_{k}\right)\right|\geq t\right\}\leq 2\exp\left(\frac{-c^{{}^{\prime \prime }}t^{2}}{16\prod_{k=1}^{n}(r_{k}∥w_{k}{∥}_{1}^{2})}\right).$$

Thus,

(A8) $$\begin{array}{ccc} P\left\{\left|\left(II\right)-2\prod_{k=1}^{n}(r_{k}∥w_{k}{∥}_{1})\right|\geq \frac{1}{2}\prod_{k=1}^{n}r_{k}{∥w_{k}∥}_{1}\right\} & \leq & 2\exp\left(\frac{-c^{{}^{\prime \prime }}}{64}\prod_{k=1}^{n}r_{k}\right). \end{array}$$

Combing (A7) and (A8), we can conclude that with probability at least

$$\begin{array}{c} 1-2\exp\left(-c^{\prime \prime }\prod_{k=1}^{n}r_{k}\right)-2\exp\left(-c^{\prime }\cdot \min\left\{\frac{\prod_{k=1}^{n}r_{k}}{\left(\prod_{k=1}^{n}(2r_{k}(∥w_{k}{∥}_{2}/∥w_{k}{∥}_{1}{)}^{2}+1)\right)-1},\right.\right.\\ \left.\left.\prod_{k=1}^{n}r_{k},\frac{\prod_{k=1}^{n}r_{k}}{\left(\prod_{k=1}^{n}(2∥w_{k}{∥}_{2}/∥w_{k}{∥}_{1}\sqrt{r_{k}\log\left(r_{k}\right)}+2∥w_{k}{∥}_{\infty }/∥w_{k}{∥}_{1}r_{k}\log\left(r_{k}\right)+1)\right)-1}\right\}\right), \end{array}$$

the following holds

$$\begin{array}{ccc} {∥H⊡\left(T-\tilde{T}\right)∥}_{F}^{2} & = & \left(I)+(II\right)\\ & \geq & 2\prod_{k=1}^{n}(r_{k}∥w_{k}{∥}_{1})-|II-2\prod_{k=1}^{n}(r_{k}∥w_{k}{∥}_{1})|-\left(I\right)\\ & \geq & \prod_{k=1}^{n}(r_{k}∥w_{k}{∥}_{1})=\left(\prod_{k=1}^{n}r_{k}\right){∥H⊡B∥}_{F}^{2}. \end{array}$$

By a union of bound over all of the points in *S*, we establish items 1 and 3 of the lemma. □

Now we are ready to prove the lower bound in Theorem 3. First we give a formal statement for the lower bound in Theorem 3 by introducing the constant $C^{\prime }$ to characterize the “flatness” of W.
**Theorem** **A7** (Lower bound for low-rank tensor when W is flat and Ω∼W). *Let $W=w_{1}⊗\cdots ⊗w_{n}\in R^{d_{1}\times \cdots \times d_{n}}$ be a CP rank-1 tensor so that all $\left(i_{1},\cdots ,i_{n}\right)\in \left[d_{1}\right]\times \cdots \times \left[d_{n}\right]$ with ${∥W∥}_{\infty }\leq 1$, so that*

$$\max_{i_{k}}|w_{k}\left(i_{k}\right)|\leq C^{\prime }\min_{i_{k}}\left|w_{k}\left(i_{k}\right)\right|,\;\mathrm{for}\;\mathrm{all}\;k=1,\cdots ,n.$$

*Suppose that we choose each $\left(i_{1},\cdots ,i_{n}\right)\in \left[d_{1}\right]\times \cdots \times \left[d_{n}\right]$ independently with probability $W_{i_{1}\cdots i_{n}}$ to form a set $\Omega \subseteq \left[d_{1}\right]\times \cdots \times \left[d_{n}\right]$. Then with probability at least 1−exp(−C·m) over the choice of Ω, the following holds:* *Let σ,β>0 and let $K_{r}\subseteq R^{d_{1}\times \cdots \times d_{n}}$ be the cone of the tensor with Tucker rank $r=[r_{1}\;\cdots \;r_{n}]$. For any algorithm $A:R^{\Omega }\to R^{d_{1}\times \cdots \times d_{n}}$ that takes as input $T_{\Omega }+Z_{\Omega }$ and outputs a guess $\hat{T}$ for T, for $T\in K_{r}\cap \beta B_{\infty }$ and $Z_{i_{1}\cdots i_{n}}∼N\left(0,\sigma^{2}\right)$, then there is some $T\in K_{r}\cap \beta B_{\infty }$ so that*

$$\begin{array}{ccc} & & \frac{∥W^{\left(1/2\right)}⊡\left(A\left(T_{\Omega }+Z_{\Omega }\right)-T\right){∥}_{F}}{∥W^{\left(1/2\right)}{∥}_{F}}\\ & \geq & c\cdot \min\left\{\frac{\beta }{\sqrt{\log(8\prod_{k=1}^{n}d_{k})}},\frac{\sigma }{\sqrt{\left|\Omega \right|}}\sqrt{\prod_{k=1}^{n}r_{k}}\cdot \min\left\{\sqrt{\frac{1}{\left(\prod_{k=1}^{n}\left(1+2C^{\prime 2}r_{k}/d_{k}\right)\right)-1}},\right.\right.\\ & & \left.\left.1,\sqrt{\frac{1}{\left(\prod_{k=1}^{n}\left(2C^{\prime }\sqrt{r_{k}/d_{k}\log\left(r_{k}\right)}+2C^{\prime }r_{k}/d_{k}\log\left(r_{k}\right)+1\right)\right)-1}}\right\}\right\}, \end{array}$$

*with probability at least $\frac{1}{2}$ over the randomness of A and the choice of Z. Above c, C are constants which depend only on $C^{\prime }$.*
**Proof.** Let $m=∥W^{\left(1/2\right)}{∥}_{F}^{2}=\prod_{k=1}^{n}{∥w_{k}∥}_{1}$, so that E|Ω|=m. We instantiate Lemma A4 with $H=W^{\left(1/2\right)}$ and B being the tensor whose entries are all 1. Let *S* be the set guaranteed by Lemma A4. We have

$$\max_{T\in S}{∥T∥}_{\infty }\leq \sqrt{\frac{1}{2}\log\left(8\prod_{k=1}^{n}d_{k}\right)\prod_{k=1}^{n}r_{k}}.$$

and

$$\max_{T\in S}∥T_{\Omega }{∥}_{F}\leq 2\sqrt{\prod_{k=1}^{n}r_{k}}{∥B_{\Omega }∥}_{F}=2\sqrt{\left|\Omega \right|\prod_{k=1}^{n}r_{k}}.$$

We also have

$$∥W^{\left(1/2\right)}⊡\left(T-T^{\prime }\right){∥}_{F}\geq \sqrt{\prod_{k=1}^{n}r_{k}}{∥W^{\left(1/2\right)}∥}_{F}=\sqrt{m\prod_{k=1}^{n}r_{k}}$$

for $T\neq T^{\prime }\in S$. Using the assumption that $w_{k}$ are flat, the size of the set *S* is bigger than or equal to

$$\begin{array}{ccc} N & = & C\exp\left(c\cdot \min\left\{\frac{\prod_{k=1}^{n}r_{k}}{\left(\prod_{k=1}^{n}(2r_{k}(∥w_{k}{∥}_{2}/∥w_{k}{∥}_{1}{)}^{2}+1)\right)-1},\prod_{k=1}^{n}r_{k},\right.\right.\\ & & \left.\left.\frac{\prod_{k=1}^{n}r_{k}}{\left(\prod_{k=1}^{n}(2∥w_{k}{∥}_{2}/∥w_{k}{∥}_{1}\sqrt{r_{k}\log\left(r_{k}\right)}+2∥w_{k}{∥}_{\infty }/∥w_{k}{∥}_{1}r_{k}\log\left(r_{k}\right)+1)\right)-1}\right\}\right)\\ & \geq & C\exp\left(c\cdot \min\left\{\frac{\prod_{k=1}^{n}r_{k}}{\left(\prod_{k=1}^{n}\left(2C^{\prime 2}r_{k}/d_{k}+1\right)\right)-1},\prod_{k=1}^{n}r_{k},\right.\right.\\ & & \left.\left.\frac{\prod_{k=1}^{n}r_{k}}{\left(\prod_{k=1}^{n}\left(2C^{\prime }\sqrt{r_{k}\log\left(r_{k}\right)/d_{k}}+2C^{\prime }r_{k}\log\left(r_{k}\right)/d_{k}+1\right)\right)-1}\right\}\right)\\ & \geq & \exp\left(C^{\prime \prime }\cdot \min\left\{\frac{\prod_{k=1}^{n}r_{k}}{\left(\prod_{k=1}^{n}\left(2C^{\prime 2}r_{k}/d_{k}+1\right)\right)-1},\prod_{k=1}^{n}r_{k},\right.\right.\\ & & \left.\left.\frac{\prod_{k=1}^{n}r_{k}}{\left(\prod_{k=1}^{n}\left(2C^{\prime }\sqrt{r_{k}\log\left(r_{k}\right)/d_{k}}+2C^{\prime }r_{k}\log\left(r_{k}\right)/d_{k}+1\right)\right)-1}\right\}\right), \end{array}$$

where $C^{\prime \prime }$ depends on *c* and *C*. Set

$$\begin{array}{c} \kappa =\min\left\{\frac{\beta }{\sqrt{\frac{1}{2}\log\left(8\prod_{k=1}^{n}d_{k}\right)\prod_{k=1}^{n}r_{k}}},\frac{\sigma \sqrt{C^{\prime \prime }}}{8\sqrt{\left|\Omega \right|}}\sqrt{\frac{\prod_{k=1}^{n}d_{k}}{\left(\prod_{k=1}^{n}\left(d_{k}+2C^{\prime 2}r_{k}\right)\right)-\prod_{k=1}^{n}d_{k}}},\frac{\sigma \sqrt{C^{\prime \prime }}}{8\sqrt{\left|\Omega \right|}},\right.\\ \left.\frac{\sigma \sqrt{C^{\prime \prime }}}{8\sqrt{\left|\Omega \right|}}\sqrt{\frac{\prod_{k=1}^{n}d_{k}}{\left(\prod_{k=1}^{n}\left(2C^{\prime }\sqrt{d_{k}r_{k}\log\left(r_{k}\right)}+2C^{\prime }r_{k}\log\left(r_{k}\right)+d_{k}\right)\right)-\prod_{k=1}^{n}d_{k}}}\;\right\}. \end{array}$$

Observe that $\frac{\sigma \sqrt{\log|S|}}{4\max_{T\in S}{∥T_{\Omega }∥}_{F}}\geq \frac{\sigma \sqrt{\log(N)}}{4\max_{T\in S}{∥T_{\Omega }∥}_{F}}$ and

$$\begin{array}{ccc} & & \frac{\sigma \sqrt{\log(N)}}{4\max_{T\in S}{∥T_{\Omega }∥}_{F}}\\ & \geq & \frac{\sigma \sqrt{C^{\prime \prime }}}{8\sqrt{\left|\Omega \right|\prod_{k=1}^{n}r_{k}}}\cdot \min\left\{\frac{\prod_{k=1}^{n}r_{k}}{\left(\prod_{k=1}^{n}\left(2C^{\prime 2}r_{k}/d_{k}+1\right)\right)-1},\prod_{k=1}^{n}r_{k},\right.\\ & & \left.\frac{\prod_{k=1}^{n}r_{k}}{\left(\prod_{k=1}^{n}\left(2C^{\prime }\sqrt{r_{k}\log\left(r_{k}\right)/d_{k}}+2C^{\prime }r_{k}\log\left(r_{k}\right)/d_{k}+1\right)\right)-1}\right\}\\ & = & \frac{\sigma \sqrt{C^{\prime \prime }}}{8\sqrt{\left|\Omega \right|}}\cdot \min\left\{\sqrt{\frac{\prod_{k=1}^{n}d_{k}}{\left(\prod_{k=1}^{n}\left(d_{k}+2C^{\prime 2}r_{k}\right)\right)-\prod_{k=1}^{n}d_{k}}},1,\right.\\ & & \left.\sqrt{\frac{\prod_{k=1}^{n}d_{k}}{\left(\prod_{k=1}^{n}\left(2C^{\prime }\sqrt{d_{k}r_{k}\log\left(r_{k}\right)}+2C^{\prime }r_{k}\log\left(r_{k}\right)+d_{k}\right)\right)-\prod_{k=1}^{n}d_{k}}}\;\right\}\geq \kappa , \end{array}$$

so this is a legitimate choice of κ in Lemma A3. Next, we verify that $\kappa S\subseteq K\cap \beta B_{\infty }$. Indeed, we have

$$\kappa \max_{S}{∥T∥}_{\infty }\leq \kappa \sqrt{\frac{1}{2}\log\left(8\prod_{k=1}^{n}d_{k}\right)\prod_{k=1}^{n}r_{k}}\leq \beta ,$$

so $\kappa S\subseteq \beta B_{\infty }$, and every element of S has Tucker rank r by construction. Then Lemma A3 concludes that if A works on $K_{r}\cap \beta B_{\infty }$, then there is a tensor $T\in K_{r}\cap \beta B_{\infty }$ so that

$$\begin{array}{ccc} & & ∥W^{\left(1/2\right)}⊡\left(A\left(T_{\Omega }+Z_{\Omega }\right)-T\right){∥}_{F}\\ & \geq & \frac{\kappa }{2}\min_{T\neq T^{\prime }\in S}{∥W^{\left(1/2\right)}⊡\left(T-T^{\prime }\right)∥}_{F}\\ & \geq & \frac{1}{2}\min\left\{\frac{\beta }{\sqrt{\frac{1}{2}\log\left(8\prod_{k=1}^{n}d_{k}\right)\prod_{k=1}^{n}r_{k}}},\frac{\sigma \sqrt{C^{\prime \prime }}}{8\sqrt{\left|\Omega \right|}}\sqrt{\frac{\prod_{k=1}^{n}d_{k}}{\left(\prod_{k=1}^{n}\left(d_{k}+2C^{\prime 2}r_{k}\right)\right)-\prod_{k=1}^{n}d_{k}}},\frac{\sigma \sqrt{C^{\prime \prime }}}{8\sqrt{\left|\Omega \right|}},\right.\\ & & \left.\frac{\sigma \sqrt{C^{\prime \prime }}}{8\sqrt{\left|\Omega \right|}}\sqrt{\frac{\prod_{k=1}^{n}d_{k}}{\left(\prod_{k=1}^{n}\left(2C^{\prime }\sqrt{d_{k}r_{k}\log\left(r_{k}\right)}+2C^{\prime }r_{k}\log\left(r_{k}\right)+d_{k}\right)\right)-\prod_{k=1}^{n}d_{k}}}\right\}\sqrt{m\prod_{k=1}^{n}r_{k}}\\ & = & \min\left\{\frac{\beta \sqrt{m}}{\sqrt{2\log(8\prod_{k=1}^{n}d_{k})}},\frac{\sigma \sqrt{C^{\prime \prime }m}}{16\sqrt{\left|\Omega \right|}}\sqrt{\prod_{k=1}^{n}r_{k}}\cdot \min\left\{\frac{1}{\sqrt{\left(\prod_{k=1}^{n}\left(1+2C^{\prime 2}r_{k}/d_{k}\right)\right)-1}},\right.\right.\\ & & \left.\left.1,\frac{1}{\sqrt{\left(\prod_{k=1}^{n}\left(2C^{\prime }\sqrt{r_{k}/d_{k}\log\left(r_{k}\right)}+2C^{\prime }r_{k}/d_{k}\log\left(r_{k}\right)+1\right)\right)-1}}\right\}\right\}. \end{array}$$

Additionally, by Lemma A2, we conclude that

$$\begin{array}{ccc} & & \frac{∥W^{\left(1/2\right)}⊡\left(A\left(T_{\Omega }+Z_{\Omega }\right)-T\right){∥}_{F}}{∥W^{\left(1/2\right)}{∥}_{F}}\\ & \geq & \tilde{c}\cdot \min\left\{\frac{\beta }{\sqrt{\log(8\prod_{k=1}^{n}d_{k})}},\frac{\sigma }{\sqrt{\left|\Omega \right|}}\sqrt{\prod_{k=1}^{n}r_{k}}\cdot \min\left\{\frac{1}{\sqrt{\left(\prod_{k=1}^{n}\left(1+2C^{\prime 2}r_{k}/d_{k}\right)\right)-1}},\right.\right.\\ & & \left.\left.1,\frac{1}{\sqrt{\left(\prod_{k=1}^{n}\left(2C^{\prime }\sqrt{r_{k}/d_{k}\log\left(r_{k}\right)}+2C^{\prime }r_{k}/d_{k}\log\left(r_{k}\right)+1\right)\right)-1}}\right\}\right\}, \end{array}$$

where $\tilde{c}$ depends on the above constants. □
**Remark** **A1.** *Consider the special case when $T\in R^{d_{1}\times d_{2}}$ with $d_{1}\leq d_{2}$. Then we can consider the reconstruction of S in Lemma A4 with $H=W^{\left(1/2\right)}$, B being the tensor whose entries are all 1, $C\in {\left\{\pm 1\right\}}^{r\times d_{2}}$, ${}^{1}U\in {\left\{\pm 1\right\}}^{d_{1}\times r}$ and ${}^{2}U\in {\left\{\pm 1\right\}}^{d_{2}\times d_{2}}$ which implies that $r_{1}=r$ and $r_{2}=d_{2}$. Thus, we have*

$$\begin{array}{ccc} & & \frac{∥W^{\left(1/2\right)}⊡\left(A\left(T_{\Omega }+Z_{\Omega }\right)-T\right){∥}_{F}}{∥W^{\left(1/2\right)}{∥}_{F}}\geq \tilde{c}\cdot \min\left\{\frac{\sigma }{\sqrt{\left|\Omega \right|}}\sqrt{rd_{2}},\frac{\beta }{\sqrt{\log(8d_{1}d_{2})}}\right\}, \end{array}$$

*which has the same bound as the one in ([19] Lemma 28).*
